# A lab-scale prototype development of Lignin-Formaldehyde (LF) resin: A bio-renewable adhesive for wood panel industries

**DOI:** 10.1371/journal.pone.0352893

**Published:** 2026-07-08

**Authors:** Arsalan Yousafzai, Tufail Rahman, Sana Ahmad, Muzaffar Iqbal, Ambar Farooq, Majid Hussain

**Affiliations:** 1 Department of Forestry and Wildlife Management, The University of Haripur, Haripur, Khyber Pakhtunkhwa, Pakistan; 2 Department of Biochemistry, Bahria University of Health Sciences, Karachi, Pakistan; 3 Department of Chemistry, The University of Haripur, Haripur, Khyber Pakhtunkhwa, Pakistan; 4 College of Material Engineering, Fujian Agriculture and Forestry University, Fuzhou, China; Galgotias University, INDIA

## Abstract

This study evaluated laboratory-prepared wood adhesives synthesized from alkaline lignins isolated from three locally available biomasses: chir pine needles (CP), eucalyptus sawdust (ESD), and sugarcane bagasse (SB). After alkaline extraction, each lignin was reacted with formaldehyde under alkaline conditions to synthesize resol-type lignin-formaldehyde resins under a complete phenol-replacement system. The Lignins differed in molecular weight, extraction yield, and hydroxyl distribution. Quantitative ^31^P NMR showed total OH + COOH contents of 3.70 mmol g^-1^ for CP, 4.60 mmol g^-1^ for ESD, and 4.83 mmol g^-1^ for SB. Thermogravimetric analysis indicated comparable thermal stability among the lignins, with char yields of 19.22–24.23% at 1000°C. The lignin formaldehyde (LF) resin showed alkaline pH, workable solid content, 3–4shear-thinning viscosity, and gel times suitable for laboratory hot pressing. Differential scanning calorimetry confirmed curing in the hot-pressing temperature range, with CP resin showing the highest glass transition temperature. In lap-shear testing, the laboratory phenol formaldehyde (PF) control showed the highest dry and wet strengths, while CP performed best among the LF resins, followed by ESD and SB. The results show that adhesive performance was not governed by total hydroxyl content alone. Instead, feedstock-dependent lignin structure, hydroxyl distribution, molecular size, and cured-network development jointly influenced bond strength and moisture resistance. The study supports the laboratory-scale potential of Pakistani biomass residues for lower-phenol wood adhesive development, while further work is needed on residual carbohydrates, panel formaldehyde emissions, commercial PF benchmarking, and scale-up feasibility.

## Introduction

Phenol-formaldehyde (PF) resins are widely used thermosetting adhesives for plywood and other wood-based panels because they provide strong bonding, good thermal stability, and high moisture resistance [1]. However, conventional PF chemistry relies on petrochemical phenol and formaldehyde, linking material cost and supply to fossil markets and motivating the development of renewable phenolic substitutes [2]. Lignin is a strong candidate phenol substitute because it is abundant, aromatic, and naturally contains phenolic motifs that can participate in PF-type condensation reactions [2,3]. Consequently, considerable attention has been paid to the use of lignin as an alternative phenol in both resol and novolac formulations [4]. The impacts of lignin on viscosity, curing rate, and bonding strength depend on the origin and type of lignin used, emphasizing the necessity of linking lignin chemistry to its properties and performance [5]. Unmodified lignins are often less reactive than phenol because reactive aromatic sites may be limited by methoxy substitution, steric hindrance, molecular size, and condensation. For this reason, previous studies have used phenolation, demethylation, fractionation, or depolymerization to improve lignin reactivity and adhesive performance at high substitution levels [2,6,7]. Lignin source is also important because syringyl/guaiacyl ratio, phenolic hydroxyl distribution, molecular weight, and condensation degree differ among softwood, hardwood, and grass lignins. These structural differences can affect formaldehyde reactivity, curing behavior, network formation, and bond strength [3,4].

Complete phenol replacement by lignin has been reported in previous resol type adhesive studies. Comparative information remains limited on how unmodified lignins from different local biomass residues perform when processed under identical extraction, synthesis, curing, and bonding conditions [3,4,6–8]. In this study, chir pine needles, eucalyptus sawdust, and sugarcane bagasse was selected as representative softwood, hardwood, and grass derived biomass sources from Pakistan. Their lignins were evaluated as the sole phenolic component in laboratory lignin formaldehyde resins to determine how feedstock dependent differences in hydroxyl distribution, molecular size, curing behavior, and network development influence adhesive performance and moisture resistance. This question is relevant for regions that generate lignocellulosic residues but still depend on petroleum-derived adhesive systems. Pakistan produces sugarcane bagasse and forest or wood-processing residues, while chir pine needles and eucalyptus sawdust are also locally available biomass resources [9–11]. These materials represent different botanical lignin types. Chir pine is expected to provide a guaiacyl-rich softwood lignin, eucalyptus a syringyl/guaiacyl hardwood lignin, and sugarcane bagasse a grass lignin with mixed syringyl, guaiacyl, and p-hydroxyphenyl character. Comparing these lignins within the same 100% phenol-replacement system clarifies how local feedstock chemistry affects resin processability, curing behavior, bond strength, and moisture resistance.

The lignins were characterized by size-exclusion chromatography, ATR-FTIR, quantitative ³¹P NMR, elemental analysis, and thermogravimetric analysis. The corresponding resins and adhesives were evaluated using pH, solids content, gel time, shear-rate-dependent viscosity, free formaldehyde content, DSC curing behavior, and dry and wet lap-shear strength. A laboratory PF control was included as a benchmark. This design allowed feedstock-specific relationships among lignin source, hydroxyl distribution, molecular size, cure behavior, and adhesive performance to be evaluated under controlled laboratory conditions.

## Materials and methods

### Raw materials

The lignocellulosic residue raw materials used in this study for lignin extraction were collected from Khyber Pakhtunkhwa province of Pakistan. Eucalyptus sawdust was collected from the sawmill in District Bannu. Sugarcane bagasse was collected from jaggery (gur) processing unit situated in the rural parts of the District Swabi. The needles of Chir pine (*Pinus roxburghii*) were collected from chir pine forest, Haripur. Fresh and clean samples were selected to reduce contamination and decomposition before processing.

### Extraction of lignin and yield determination

The extraction of lignin involved alkali solubilization and acid precipitation. Lignin has an inherent tendency towards being soluble in alkali solutions and precipitable in acidic environments. This technique has been employed extensively on sugarcane and wood wastes without destroying the valuable phenolic compounds for resin production [12,13]. For each biomass, 10 g of dried and sieved powder was treated with 0.5 M NaOH at 70ºC for 3 h under continuous stirring. The mixture was filtered, and the filtrate was acidified to approximately pH 1 using 1 M H_2_SO_4_ to precipitate lignin. The precipitated lignin was recovered by centrifugation at 5000 rpm for 15 min, washed thoroughly with distilled water, and dried at 80ºC to constant weight before storage [8,14–16].

The dissolution, filtration, washing and acid precipitation steps also helped remove soluble sugars and inorganic salts that could affect lignin reactivity and resin curing. The washed precipitates were dried to constant weight and storage in airtight containers before characterization and resin preparation. Lignin yield was calculated gravimetrically as the percentage of dried lignin recovered from the initial dry biomass mass. Residual carbohydrate content was not directly quantified in this study. Therefore, possible carbohydrate effects were considered as a limitation and interpreted indirectly using ash content, elemental composition, FTIR, and quantitative ³¹P NMR results.

### Physiochemical, elemental, and molecular-weight analysis of lignin

The moisture content of extracted lignin samples was determined gravimetrically. Approximately 1 g of each lignin was dried in a hot-air oven at 80°C for 3 hours until constant weight was achieved. Moisture content was calculated as:


$$\begin{array}{c} Moisture\;(\%)=\frac{\left(initial\;mass-dry\;mass\right)}{initial\;mass}\times \text{1}\text{0}\text{0} \end{array}$$ (1)

Ash content was determined according to the TAPPI T 211 om-93. Approximately 0.5 g of oven-dried lignin was weighed in a pre-weighed ceramic crucible and converted to ash in a muffle furnace at 525 ± 25°C for 4 h. Temperature was first raised gradually from approximately 250°C to reduce flaming. The crucible was cooled in a desiccator and weight [17]. Ash content was calculated as:


$$\begin{array}{c} Ash\;(\%)=\frac{mass\;of\;ash}{initial\;dry\;mass}\times \text{1}\text{0}\text{0} \end{array}$$ (2)

Elemental composition of lignin samples was determined by CHNS combustion with thermal conductivity detection. This analysis was performed using a Euro EA elemental analyzer (Euro Vector, Italy). Oxygen content was calculated by difference after accounting for measured C, H, N, moisture, and ash. The elemental results were used to compare lignins and to support interpretation of functional-group availability relevant to thermoset resin formulation [8,18].

Molecular-weight distribution (Mn, Mw) and polydispersity index (PDI) were measured by size-exclusion chromatography (SEC) using an Agilent 1200 HPLC/GPC system with UV detection at 280 nm. Lignin (10 mg) was dissolved in HPLC-grade THF (10 mL) for 24 h at room temperature and filtered through a 0.45 µm PTFE syringe filter. Separation was performed isocratically with THF at 0.5 mL min^-1^ (injection volume 200 µL; sample concentration 1 mg mL^-1^). Calibration used polystyrene standards (1,000–100,000 Da); therefore, the reported Mn and Mw values are relative (polystyrene-equivalent) and are used here for comparative assessment across lignin sources [8,19].

### ATR-FTIR and quantitative ^31^P NMR analysis

FTIR was performed in ATR mode to compare functional groups across lignin sources. Lignin samples were dried at 80°C for 3 h before analysis, and approximately 1−2 mg was placed directly on a diamond ATR crystal. Spectra were collected from 4000 to 400 cm^-1^ at 4 cm^-1^ resolution with 32 scans per sample. ATR-FTIR is suitable for technical lignins and avoids pellet-preparation artifacts [8]. FTIR was used for qualitative functional-group comparison and not for quantitative carbohydrate determination.

Quantitative ^31^P NMR was used to determine hydroxyl-group distributions. Approximately 40 mg of vacuum-dried lignin was dissolved in 0.5 mL of dry anhydrous pyridine/CDCl_3_ at a 1.6:1 volume ratio. Chromium (III) acetylacetonate, Cr (arcac)_3_ was added as the relaxation agent at 0.05% (w/v). A 0.10 mL aliquot of cyclohexanol solution was used as the internal standard, followed by addition of 0.05 mL of 2-chloro-4,4,5,5-tetramethyl-1,3,2-dioxaphospholane under dry nitrogen. The mixture was allowed to react for 15 min and then transferred to a sealed 5 mm NMR tube. Spectra were acquired using a Bruker AVANCE III HD 600 MHz NMR spectrometer with an approximate ³¹P frequency of 242.9 MHz at 298 K. Acquisition used a 90° pulse angle, 10 s relaxation delay, inverse-gated proton decoupling, 256 scans, and a 20-ppm spectral window covering 152–132 ppm. Cyclohexanol was used as the internal reference at 145.15 ppm. Spectra were processed by line broadening, zero filling, phase correction, baseline correction, chemical-shift calibration, and region-wise integration [20,21].

### TGA analysis of lignin samples

To determine the thermal stability of the oven-dried lignins from chir pine (CP), eucalyptus sawdust (ESD), and sugarcane bagasse (SB), thermogravimetric analysis was conducted using a TA Instruments TGA Q50. Experiments were performed under flowing nitrogen to prevent oxidation degradation. Samples were loaded into a platinum crucible and heated from 25 to 1000°C at a rate of 10°C min^-1^ with a nitrogen flow rate of 20 mL min^-1^. The instrument was calibrated for temperature and mass before analysis. Mass-loss and derivative thermogravimetric curves were used to determine thermal-degradation behavior and char yield. Analyses were performed in duplicate for each lignin samples [4,8,22].

### Synthesis of LF resin, laboratory PF control, and adhesive formulation

This study reports LF resin prepared with complete phenol replacement by lignin. Earlier lower-substitution trials were not included in the present dataset. For LF synthesis, the lignin-to-formaldehyde formulation followed the same general molar basis used for PF-type resol preparation, following Kalami et al. [8]. Initially, lignin was dissolved in 1 M NaOH and then added to formaldehyde (37%) in a three-necked flask. The flask temperature was controlled using a dry bath heating system. The temperature was raised to 65°C within 30 min and kept at that point for another 10 min before adding the remaining amount of NaOH solution (approximately 1/3 of the initial amount). The temperature was then gradually increased to 85°C and held constant for 1 h under agitation at 110 rpm with coated magnetic stir bars. The mixture was cooled to ambient temperature, and the resin was stored at −18°C to limit further polymerization [8,23]. A laboratory PF control was prepared using the same general resol-type procedure, except that phenol was used as the phenolic component instead of lignin. The PF control was included as a laboratory benchmark for resin properties and lap-shear performance. It was not treated as a commercial PF adhesive.

LF adhesive was prepared using the same adhesive-mixing procedure for all lignin sources. Wheat flour (6.5%, by mass) and alder bark material (6.5%, by mass) were slowly added to water (18%, by mass) and mixed until dispersed. Thawed LF resin (66%, by mass) and NaOH (3%, by mass) were then added, and the mixture was agitated using with an overhead digital mixer at 300 rpm. Wheat flour was used as an extender and rheology modifier to improve spread ability, reduce excessive penetration into the veneer, and support uniform bond-line formation. Alder bark material was used as a lignocellulosic filler to increase bond line body and reduce resin loss into porous wood substrate [19,23]. Both additives were kept constant in all formulations; therefore, differences in adhesive performance were interpreted mainly in relation to lignin source rather than additive loading.

### Resin, adhesive and free formaldehyde characterization

The nonvolatile (solid) content of both the commercial and synthesized lignin-based resins was measured according to ASTM D4426-01. For this test, approximately 1.0–2.0 g of liquid resin was placed in a vented oven set at 135°C for 2 h. After heating, the sample was removed and allowed to cool in a desiccator at room temperature, then weighed again. The nonvolatile content was calculated as the percentage of the remaining mass relative to the original mass of the resin. In order to minimize excessive penetration into wood substrates and forecast the final adhesive bond-line thickness, this test estimates the resin’s solid percentage [23]. The viscosity of each liquid resin was measured at 23°C ± 1°C using a rotational rheometer. Measurements were collected over shear rates of 1–1000 s ⁻ ¹ to evaluate shear-rate dependence and to address possible 3–4shear-thinning behavior. Lower shear rates were included for formulation comparison, while the higher shear rate was retained to represent spreading-related flow during adhesive application. Viscosity is important because excessive viscosity can reduce wetting and spreading, whereas very low viscosity can cause over-penetration and starved bond lines [19]. The pH of each liquid resin was measured using a digital benchtop pH meter with a glass electrode. The electrode was calibrated with standard buffer solutions at pH values of 4.0, 7.0, and 10.0 prior to the measurement. Resin pH is important because it affects storage stability, curing rate, and resol-type condensation reaction [23,24].

Gel time was determined by placing approximately 1 g of resin into a glass tube immersed in boiling water at 100°C. The time required for gel-strand formation was recorded. Gel time provides a rapid indication of resin reactivity during thermal polymerization [23,24]. Free formaldehyde content was determined for the laboratory PF control and LF resins following the titration-based method used by Kalami et al. [8]. Results were calculated from three replicate measurements and reported as mean ± SD. These measurements represent free formaldehyde in liquid resin and should not be interpreted as formaldehyde emission from finished wood panels. Differential scanning calorimetry was used to evaluate thermal curing behavior. Approximately 5 mg of resin was loaded into an aluminum pan. The instrument was calibrated using indium and zinc standards. Tests were performed under nitrogen atmosphere at approximately 20 mL min^-1^. Samples were heated from 25 to 300°C at 10°C min^-1^. Thermograms were used to determine curing onset temperature, peak temperature, enthalpy, and glass transition behavior relevant to hot pressing [25,26].

### Lap shear samples preparation and testing

Bonding strength was assessed using single-lap joints prepared from poplar wood veneers. Specimen geometry followed the principles of ASTM D906 and ISO 4587 for single-lap joints [23]. Veneer strips were approximately 25.4 mm wide, 102 mm long and 5.6 mm thick. Adhesive was applied to a 25 mm × 25 mm overlap area, corresponding to 625 mm^2^, using approximately 0.10 g adhesive per joint. The assembled joints were cured in a laboratory hot press at approximately 1400 kPa and 180°C for 3–4 min. Samples were cooled under light contact pressure and conditioned for at least 24 h at approximately 25°C and 60% relative humidity before testing [8,23]. The press temperature was selected to exceed the DSC curing peak temperature of the LF resins, thereby supporting cure within the selected press cycle [8].

Dry lap-shear strength was determined using a universal testing machine fitted with a lap-shear fixture at a crosshead speed of 5 mm min^-1^. The maximum load at failure was divided by the bonded area to calculate shear strength. Three replicate specimens were tested for each adhesive system [8]. For wet-strength testing, specimens were aged following ASTM D3434 principles by boiling at 100°C for 4 h, conditioning at 65°C for 20 h, and boiling again for 4 h before mechanical testing. This cyclic aging procedure was used to evaluate moisture resistance of the cured adhesives bonds [27,28].

### Statistical analysis

A two-way ANOVA was used to evaluate the effects of LF resin type and conditioning state on lap-shear strength. Tukey HSD post hoc comparisons were applied at p < 0.05. Three replicates’ specimens were included for each LF resin and conditioning state [8,27]. The laboratory PF control was reported as a benchmark and was not included in the LF-only two-way ANOVA because the main statistical objective was to compare the three lignin feedstocks under dry and wet conditioning.

## Results and discussion

### Lignin yield, physicochemical properties, elemental composition, and molecular weight

Lignin yield differed among the three biomass sources. CP gave the highest yield (15.13 ± 0.21%), followed by ESD (13.07 ± 0.38%) and SB (10.77 ± 0.31%). These results show that all three residues provided recoverable alkaline lignin under the selected extraction conditions, but feedstock type affected extraction efficiency. This point is important for process feasibility because a lignin source with acceptable adhesive performance but low recovery may be less attractive for scale-up. Ash content, which reflects remaining inorganic impurities, was 2.37% for CP, 1.90% for ESD, and 1.47% for SB (Table 1). Ash content in adhesives should be minimal since salt may interfere with the resin curing process, as well as increase viscosity while in storage. All ash contents found are less than 3%, which is usually acceptable for phenolic-type adhesives provided that the washing is effective [8]. The low ash values suggest that washing reduced inorganic residues, although they do not prove complete removal of organic impurities. Oxygen content can vary with lignin functionality and possible residual carbohydrate contribution; therefore, oxygen values were interpreted as comparative indicators rather than direct purity measurements.

**Table 1:** Lignin yield, physicochemical properties, molecular-weight distribution, and elemental analysis of lignins extracted from chir pine (CP), eucalyptus sawdust (ESD), and sugarcane bagasse (SB).

Lignin Samples	Yield (%)	Moisture content %	Ash content %	Mn g mol^-1^	Mw g mol^-1^	PDI = Mw/Mn	Elemental analysis (%)			
							C	H	N	O
Chir Pine (CP) Lignin	15.13 ± 0.21	4.9	2.37	7751	11342	1.46	44.5	5.6	0.5	42.13
Eucalyptus Sawdust (ESD) Lignin	13.07 ± 0.38	3.21	1.9	3275	4285	1.3	45.2	5.9	0.6	43.19
Sugarcane Bagasse (SB) Lignin	10.77 ± 0.31	3.72	1.47	2160	2891	1.34	44.0	5.8	0.7	44.31

Values for lignin yield are mean ± SD (n = 3). Mn, number-average molecular weight; Mw, weight-average molecular weight; PDI, polydispersity index. Oxygen was calculated by difference.

The elemental analysis indicated almost similar carbon and hydrogen contents for all samples at around 44–45 wt% C and 5.6–5.9 wt% H (see Table 1). The nitrogen content was very low (0.5-0.7 wt%), which is an advantage since nitrogen is known to give rise to possible side reactions and color formation when used in excess amounts in alkaline thermosetting systems. The oxygen content (by difference) was quite high (around 42–44 wt%). The presence of such oxygen characteristics was anticipated in lignin, indicating an abundance of hydroxyl and ether functional groups that are crucial in forming resins and building networks [29]. Residual carbohydrate composition was not quantified; therefore, lignin purity and carbohydrate-related effects were not directly confirmed and were considered a study limitation.

There were distinct variations in molecular weight among the three types of lignin based on the results obtained using size exclusion chromatography (Table 1). Lignin extracted from CP had the highest Mn and Mw (Mn = 7751 g mol^-1^; Mw = 11342 g mol^-1^), with a PDI of 1.46. The molecular weight of lignin extracted from ESD was intermediate (Mn = 3275 g mol^-1^; Mw = 4285 g mol^-1^; PDI = 1.30). The molecular weights for SB lignin were the smallest (Mn = 2160g mol^-1^; Mw = 2891 g mol^-1^; PDI = 1.34). Molecular weights derived from SEC are relative measurements based on the calibration of polystyrene in THF. Lower molecular weight can improve mobility and accessibility during resin synthesis, but it does not alone guarantee better adhesive performance. This was important in the present study because SB had the lowest molecular weight but did not show the highest bond strength. The relatively narrow PDI range (1.30–1.46) indicates moderate distribution breadth among the extracted lignins.

### ATR-FTIR characterization of lignins

**Fig 1:**
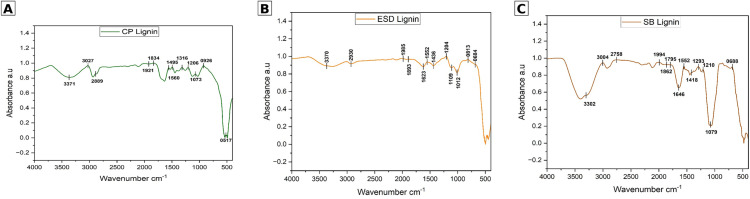
ATR-FTIR spectra of lignins extracted from (A) chir pine (CP), (B) eucalyptus sawdust (ESD), and (C) sugarcane bagasse (SB). Spectra were collected in ATR mode over 4000–400 cm⁻¹ at 4 cm⁻¹ resolution using 32 scans per sample.

ATR-FTIR was used to compare major functional groups and botanical differences in CP, ESD, and SB lignins (Table 2; Fig 1). Broad O-H stretching appeared between 3300 and 3370 cm ⁻ ¹, indicating hydrogen-bonded hydroxyl groups. Aromatic skeletal vibration in the 1600−1500 cm^-^1 region supported the presence of lignin aromatic structures [30]. Bands in the 1200−1000 cm^-1^ region were assigned mainly to aryl˗O and C˗O stretching, although this region may also include contributions from residual polysaccharides [30,31]. Therefore, FTIR was interpreted qualitatively and not used to quantify carbohydrate impurities.

**Table 2:** FTIR assignment of bands for lignins from chir pine (CP), eucalyptus sawdust (ESD), and sugarcane bagasse (SB).

Assignment	Wavenumbers (cm ⁻ ¹)		
	CP	ESD	SB
O-H stretching (hydroxyl groups, H-bonded)	3371.38	3369.51	3302.42
CH stretching (aromatic = C-H)	3026.6	—	3004.23
Aliphatic C-H stretching (methyl, methylene groups)	2888.69	2929.73	—
Weak C-H region band, not used as diagnostic	—	—	2758.23
Weak high wavenumber band, not used for structural interpretation	—	—	1994.13
Weak overtone/combination-region band, not used as primary diagnostic evidence	1921.44	1984.81	1861.81
Weak overtone/combination-region band, not used as primary diagnostic evidence	1833.85	1893.49	1794.71
Aromatic skeletal vibrations (C = C, conjugated)	1559.89	1623.17	1645.62
Aromatic skeletal vibrations (C = C)	1494.66	1552.44	1552.44
C-H deformation (methyl, methylene groups)	—	1435.52	1418.25
C-O stretching (aryl-O), aromatic skeletal	1315.75	—	1293.39
C-O stretching (syringyl + guaiacyl units)	1205.79	1203.93	1209.52
C-O stretching (secondary alcohols, ethers)	1073.47	1108.88	1079.06
C-O stretching; possible polysaccharides contribution	—	1011.58	—
C-H out-of-plane (aromatic ring vibration)	926.24	—	—
Aromatic C-H deformation (para-substituted)	—	812.56	—
C-H out-of-plane (aromatic bending, weak band)	—	683.97	687.69
low-frequency aromatic ring deformation	517.42	—	—

Note: Weak bands in the 2000–1750 cm⁻¹ region were treated as overtone/combination-region signals and were not used as primary evidence for lignin classification. Major lignin interpretation was based on the established O–H, aromatic skeletal, aryl–O, C–O, and syringyl/guaiacyl-related bands [30, 31].

For CP lignin, the spectrum (Fig 1) showed peaks at 3371.38 cm ⁻ ¹ (O-H), 3026.60 cm ⁻ ¹ (aromatic C-H), and 2888.69 cm ⁻ ¹ (aliphatic C-H). Aromatic skeletal vibrations were observed in the mid-region. The fingerprint region comprised vibrations associated with aryl-O and C-O stretching vibrations. CP showed a band at 926.24 cm^-1^, which is consistent with guaiacyl-rich softwood lignin features (Table 2). But this band was interpreted together with the broader aromatic and C-O fingerprint region rather than a single diagnostic marker. This interpretation follows FTIR-based lignin classifications that distinguish G-type softwood lignins from GS-type hardwood and herbaceous lignins [30].

Regarding ESD lignin, peaks were observed for the O-H stretching vibration at 3369.51 cm ⁻ ¹ and the aliphatic C-H stretch at 2929.73 cm ⁻ ¹, while the skeletal vibration appeared at 1623.17 and 1552.44 cm ⁻ ¹ (Fig 1 and Table 2). The fingerprint region showed bands at 1203.93 and 1108.88 cm ⁻ ¹, which are consistent with the syringyl/guaiacyl related C-O vibrations commonly observed in hardwood GS lignins [30,31]. The presence of syringyl-related bands supports the hardwood character of ESD lignin, although FTIR alone cannot quantify syringyl content [29].

The O-H and aromatic bands of SB lignin have been found to appear at 3302.42 cm ⁻ ¹, 1645.62 cm ⁻ ¹, and 1552.44 cm ⁻ ¹ from Fig 1 and Table 2. SB showed fingerprint-region bands at 1293.39, 1209.52, and 1079.06 cm ⁻ ¹, consistent with aryl-O and C-O stretching modes expected in grass-derived lignin. Overall, the FTIR patterns support the expected botanical trend: CP showed features consistent with guaiacyl-rich softwood lignin, whereas ESD and SB showed mixed syringyl/guaiacyl features typical of hardwood and herbaceous lignins. However, these assignments were considered qualitative and were supported by ^31^P NMR rather than used alone for structural confirmation [30,31].

### Quantitative ^31^P NMR analysis

**Fig 2:**
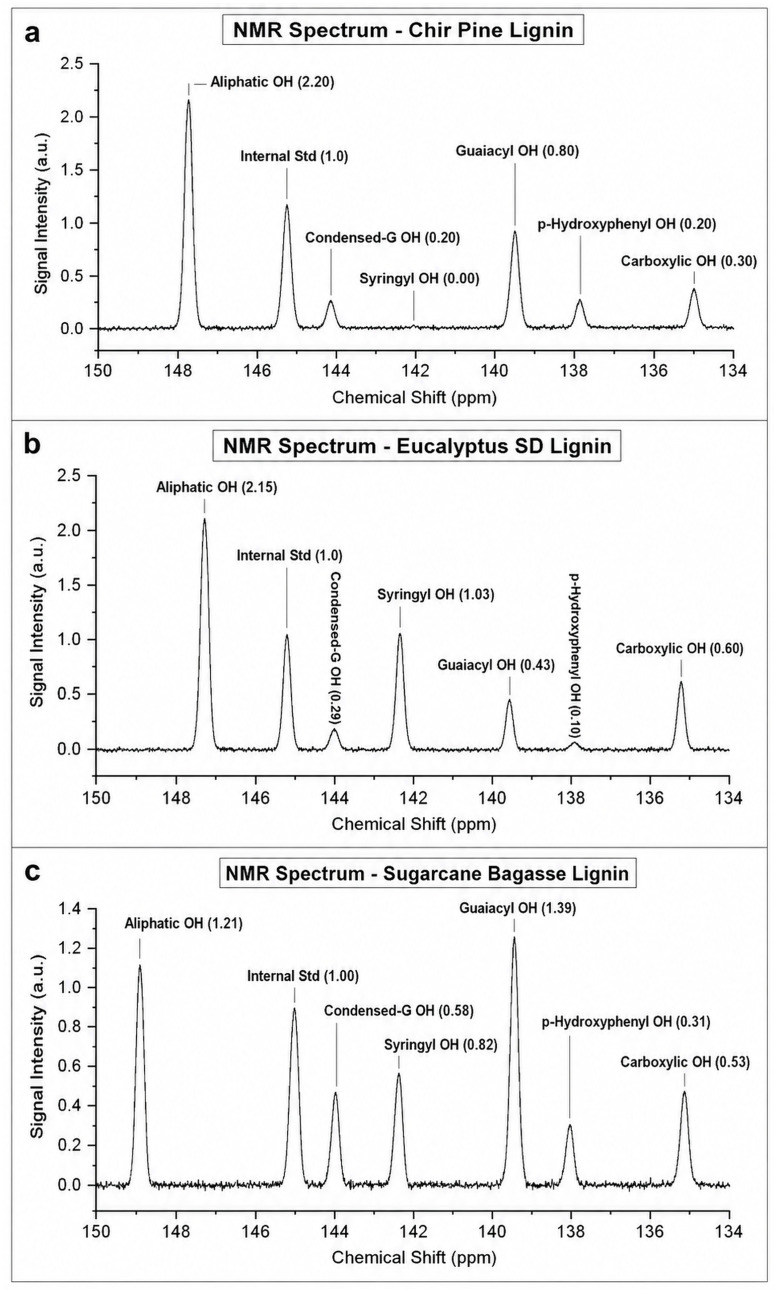
Quantitative ³¹P NMR spectra of lignins extracted from (A) chir pine (CP), (B) eucalyptus sawdust (ESD), and (C) sugarcane bagasse (SB). Approximately 40 mg of vacuum-dried lignin was derivatized with TMDP in anhydrous pyridine/CDCl₃ using cyclohexanol as the internal standard. Spectra were acquired at 298 K using inverse-gated proton decoupling and a 10 s relaxation delay.

Quantitative ³¹P NMR directly measures hydroxyl groups that influence methylolation and subsequent condensation during lignin-formaldehyde resin synthesis [20,32,33]. The integration results showed distinct hydroxyl distributions among CP, ESD, and SB lignins (Table 3; Fig 2).

**Table 3:** ^31^P NMR hydroxyl content of lignins from chir pine (CP), eucalyptus sawdust (ESD), and sugarcane bagasse (SB).

Region	Chemical shift (ppm)	CP (mmol g^-1^)	ESD (mmol g^-1^)	SB (mmol g^-1^)
Aliphatic OH	149.1-145.4 / 147.25	2.20	2.15	1.21
Condensed-G OH	144.6-143.3 / 144.00	0.20	0.29	0.58
Syringyl OH	143.3-142.0 / 142.65	0.00	1.03	0.82
Guaiacyl OH	140.5-138.6 / 139.55	0.80	0.43	1.39
p-Hydroxyphenyl OH	138.5-137.3 / 137.90	0.20	0.10	0.31
Carboxylic OH	135.9-134.0 / 135.00	0.30	0.60	0.53
Total phenolic OH	—	1.20	1.85	3.09
Total OH + COOH	—	3.70	4.60	4.83

**Note:** Total phenolic OH was calculated as the sum of condensed-guaiacyl, syringyl, guaiacyl, and p-hydroxyphenyl OH groups. Total OH + COOH was calculated as aliphatic OH + total phenolic OH + carboxylic OH.

Total OH + COOH followed the order SB (4.83 mmol g ⁻ ¹)> ESD (4.60 mmol g ⁻ ¹)> CP (3.70 mmol g ⁻ ¹). CP lignin contained aliphatic OH of 2.20 mmol g ⁻ ¹ and guaiacyl OH of 0.80 mmol g ⁻ ¹, with no measurable syringyl OH. This profile is consistent with guaiacyl-rich softwood lignin. ESD lignin showed a strong syringyl OH contribution of 1.03 mmol g ⁻ ¹ and the highest carboxylic OH value of 0.60 mmol g ⁻ ¹, supporting its hardwood-type character. SB lignin showed mixed S/G/H character, with guaiacyl OH as the dominant phenolic contribution and the highest total phenolic OH value of 3.09 mmol g ⁻ ¹. These results are important for interpreting adhesive performance because the highest hydroxyl content did not correspond to the highest bond strength. SB lignin showed the highest total OH + COOH and total phenolic OH, but its resin produced lower lap-shear strength than CP. This indicates that adhesive performance was not governed by hydroxyl quantity alone. In LF systems, the effectiveness of hydroxyl groups depends on their type, position, accessibility, and ability to participate in methylolation and subsequent methylene-bridge formation. Therefore, lignin molecular size, condensation degree, steric accessibility of aromatic reactive sites, and crosslinking efficiency must be considered together with total hydroxyl content. This interpretation agrees with previous lignin-phenolic adhesive studies showing that lignin source, molecular architecture, and functional-group distribution strongly influence curing behavior and bond performance [3,8,22,34].

### Thermogravimetric analysis of extracted lignin

**Fig 3:**
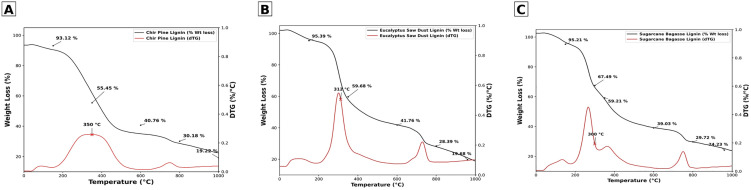
TGA and DTG curves of lignins extracted from (A) chir pine (CP), (B) eucalyptus sawdust (ESD), and (C) sugarcane bagasse (SB). Measurements were performed under nitrogen from 25 to 1000°C at a heating rate of 10°C min⁻¹ and nitrogen flow of 20 mL min⁻¹.

All three lignins showed multistage thermal degradation under nitrogen, consistent with reported lignin thermal degradation behavior [35] (Fig 3). CP retained 93.12% mass at 150°C, 55.45% at 350°C, 40.76% at 600°C, and 30.18% at 800°C, with a final char yield of 19.22% at 1000°C. ESD retained 95.39% at 150°C, 59.68% at 350°C, 41.76% at 600°C, and 28.39% at 800°C, with 19.68% char at 1000°C. SB retained 95.21% at 150°C, 59.21% at 350°C, 39.03% at 600°C, and 29.72% at 800°C, with the highest final char yield of 24.23% at 1000°C. The DTG maximum occurred at approximately 350°C for CP, 312°C for ESD, and 300°C for SB. These results indicate that all lignins had sufficient thermal stability for the hot-pressing conditions used in this study. The higher char yield of SB suggests greater formation of thermally stable carbonaceous residue during heating, although this did not correspond to superior adhesive performance.

### Resin pH, gel time, and PF comparison

All LF resins were alkaline with pH values of 10.1 for CP resin, 9.7 for ESD, and 9.5 for SB (Table 4). The laboratory PF control had a pH of 10.37. Alkaline pH supports hydroxymethylation and condensation in resol-type systems and is consistent with lignin-phenol-formaldehyde resin chemistry [23,24,34].

**Table 4:** Physicochemical analysis of LF resins and laboratory PF control.

Resin	pH	Solid content (%)	Gel time (s)	Free formaldehyde (%)
CP LF resin	10.1	49.2	360	6.43 ± 0.15
ESD LF resin	9.7	46.7	390	6.67 ± 0.06
SB LF resin	9.5	44.9	480	6.73 ± 0.21
Laboratory PF control	10.37	49.63	299.33	5.93 ± 0.25

Viscosity decreased with increasing shear rate for all resins, confirming shear-thinning behavior (Table 5). At 1 s ⁻ ¹, viscosities were 0.580 Pa·s for CP, 0.620 Pa·s for ESD, 0.570 Pa·s for SB, and 0.634 Pa·s for PF. At 1000 s ⁻ ¹, the corresponding values decreased to 0.335, 0.365, 0.350, and 0.376 Pa·s. The lower-shear-rate data show clearer differences among formulations than the single high-shear-rate values. This addition addresses the concern that viscosity measured only at 1000 s ⁻ ¹ could mask formulation-dependent differences. From an application perspective, the observed shear-thinning behavior is useful because higher viscosity at low shear can help maintain adhesive body, while lower viscosity under spreading shear can improve flow and wetting [19].

**Table 5:** Viscosity of LF resins and laboratory PF control measured at 23°C over multiple shear rates.

Shear rate (s ⁻ ¹)	CP resin (Pa·s)	ESD resin (Pa·s)	SB resin (Pa·s)	PF resin (Pa·s)
1	0.58	0.62	0.57	0.634
5	0.52	0.56	0.52	0.573
10	0.49	0.53	0.49	0.541
50	0.44	0.48	0.45	0.491
100	0.41	0.44	0.42	0.455
500	0.36	0.39	0.37	0.401
1000	0.335	0.365	0.35	0.376

Solid content followed the order Cp (49.2%)> ESD (46.7%)> SB (44.9%), while the laboratory PF control showed 49.63% solid (Table 4). Gel time increased from CP (360 s) to ESD (390 s) and SB (480 s), whereas the PF control gelled faster at 299.33 s... The longer gel times of LF resin indicate slower network formation compared with the PF control. The difference is expected because lignin contains fewer accessible reactive aromatic sites than phenol and has greater structural heterogeneity [2,8,22,34]. Free formaldehyde content was lowest in the laboratory PF control (5.93 ± 0.25%) and slightly higher in the LF resins, increasing from CP (6.43 ± 0.15%) to ESD (6.67 ± 0.06%) and SB (6.73 ± 0.21%) (Table 4). This trend suggests that complete phenol replacement with unmodified lignin did not reduce residual free formaldehyde under the present synthesis conditions [8]. The values represent free formaldehyde in liquid resin, not formaldehyde emission from finished wood panels. Therefore, these results should be interpreted as a resin-level chemical indicator, and panel emission testing remains necessary before environmental or industrial emission claims can be made.

### DSC analysis of resins

**Fig 4:**
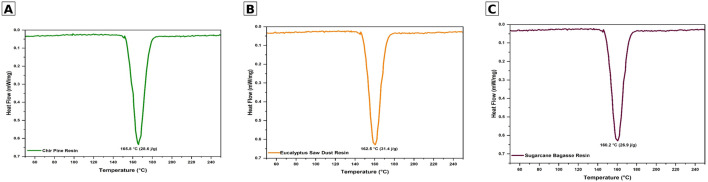
DSC thermograms of lignin-formaldehyde resins prepared from (A) chir pine (CP), (B) eucalyptus sawdust (ESD), and (C) sugarcane bagasse (SB) lignins. Approximately 5 mg of sample was analyzed under nitrogen from 25 to 300°C at a heating rate of 10°C min⁻¹.

All LF resins showed clear exothermic curing events in the DSC thermograms (Fig 4).

CP resin showed a curing onset at 140.2°C, a peak at 165.8°C, an enthalpy of 28.6 J g ⁻ ¹, and the highest Tg value of 156.3°C (Table 6). ESD resin showed the earliest onset at 135.8°C, a peak at 162.5°C, and the highest enthalpy of 31.4 J g ⁻ ¹. SB resin showed an onset at 138.0°C, the lowest peak temperature at 160.2°C, an enthalpy of 26.9 J g ⁻ ¹, and the lowest Tg value of 150.5°C. These results show that ESD began curing earlier, SB reached its main curing peak at the lowest temperature, and CP produced the most thermally rigid cured network. The higher Tg of CP resin suggests more effective network development under the selected curing conditions.

**Table 6:** DSC curing parameters of LF resins.

Sample	Tg (°C)	Tonset/Tpeak (°C)	ΔH (J g^-1^)
CP Resin	156.3	140.2 / 165.8	28.6
ESD Resin	152.7	135.8 / 162.5	31.4
SB Resin	150.5	138.0 / 160.2	26.9

The curing peak range of 160.2–165.8°C was compatible with the selected hot-press temperature of 180°C. When the DSC data are interpreted together with the ³¹P NMR results, total hydroxyl abundance alone does not explain resin behavior. SB had the highest total OH + COOH value, but it showed the longest gel time and the lowest Tg among the LF resins. In contrast, CP had the lowest total OH + COOH value but showed the shortest gel time and highest Tg. This suggests that CP lignin formed a more effective cured network under the selected alkaline synthesis and hot pressing conditions. The improved curing response may be related to better accessibility of reactive guaiacyl sites and more favorable network development. Whereas the higher hydroxyl content of SB may include hydroxyl groups that increase hydrophilicity without proportionally increasing crosslink density. Thus, hydroxyl distribution, reactive site accessibility, molecular architecture, and crosslinking efficiency were more decisive than total hydroxyl quantity alone [3,8,22,34].

### Lap-shear strength and moisture resistance

The lap-shear data showed that all LF formulations formed measurable bonds under the selected laboratory hot-pressing condition (Table 7). The laboratory PF control gave the highest dry and wet strengths, as expected for a phenol-based resol system. Among the LF resins, CP consistently showed the highest dry and wet strength, followed by ESD and SB. This strength order support the ^31^P NMR and DSC interpretation that effective network formation, rather than total hydroxyl abundance alone, controlled adhesive performance. This trend suggests that CP lignin produced a more effective cured network under the present synthesis, formulation, and pressing conditions. However, the LF resins should be interpreted as laboratory prototypes rather than industrial equivalents, because performance depends on substrate, adhesive spread, additives, pressing conditions, and resin chemistry [8,27,28,34].

**Table 7:** Dry and wet lap-shear strength of LF adhesive bonds and laboratory PF control.

Adhesive type	Lap area (mm²)	Dry shear strength (MPa)	Wet shear strength (MPa)	Wet/Dry retention (%)	Failure mode
CP LF	625	3.12 ± 0.01	1.92 ± 0.02	61.5	Cohesive Failure
ESD LF	625	2.85 ± 0.02	1.69 ± 0.01	59.3	Cohesive Failure
SB LF	625	2.34 ± 0.02	1.46 ± 0.01	62.4	Cohesive Failure
Laboratory PF control	625	3.40 ± 0.03	2.31 ± 0.02	67.9	Cohesive Failure

Values are mean ± SD (n = 3). PF control values are presented as a laboratory benchmark.

The laboratory PF control showed dry and wet strengths of 3.40 ± 0.03 MPa and 2.31 ± 0.02 MPa, respectively. Among the LF resins, dry strength decreased in the order CP (3.12 ± 0.01 MPa)> ESD (2.85 ± 0.02 MPa)> SB (2.34 ± 0.02 MPa). The same order was observed after wet conditioning, with values of 1.92 ± 0.02 MPa for CP, 1.69 ± 0.01 MPa for ESD, and 1.46 ± 0.01 MPa for SB (Table 7). Wet/dry strength retention was 67.9% for the PF control, 61.5% for CP, 59.3% for ESD, and 62.4% for SB. The decrease in wet strength agrees with previous lignin-based adhesive studies showing that moisture exposure can weaken the wood–adhesive interface and less densely crosslinked regions of the cured network [27,28,34]. However, direct comparison with published strength values should be made cautiously because adhesive performance depends on lignin type, lignin modification, substrate, additive loading, pressing schedule, and test method. In the present study, CP approached the laboratory PF benchmark more closely than ESD and SB, but the lower wet/dry retention compared with PF shows that moisture durability remains a limitation of the LF prototypes. Increasing replicate numbers, wood substrates, commercial PF benchmarking, and panel-scale tests would strengthen future validation.

The wet-strength reduction shows that the current LF systems require further optimization for applications exposed to severe moisture conditions. Possible improvement routes include lignin activation, adjustment of formaldehyde-to-lignin ratio, optimization of NaOH level, modified filler loading, longer or optimized pressing schedules, and incorporation of additional bio-based crosslinking strategies [6,7,28,34]. Recent natural material and process optimization studies further show that final material performance is strongly affected by formulation variables, processing conditions, and application-specific testing. Therefore, LF adhesive development should include structured optimization rather than single-factor changes [36–39]. These approaches should be evaluated together with free formaldehyde and panel-emission testing to avoid improving strength at the expense of environmental performance.

### Statistical analysis

Two-way ANOVA was applied to the LF resin data to evaluate the effects of lignin source and conditioning state on lap-shear strength. Resin type, conditioning, and their interaction were all significant (Table 8; p < 0.0001). The significant resin effect shows that feedstock-dependent lignin chemistry influenced cured-bond performance. The significant conditioning effect confirms the expected strength loss after wet aging. The significant resin × condition interaction indicates that wet conditioning did not affect all LF formulations equally, supporting the view that cured-network structure and moisture resistance differed among lignin sources [40,41]. Tukey HSD comparisons separated all LF resin-condition means into distinct groups (Table 9). The performance order was CP > ESD > SB in both dry and wet states. This order agrees with the shorter gel time and higher Tg of CP resin, but it does not follow total OH + COOH content alone. Therefore, the statistical results support the mechanistic interpretation that bond strength depended on lignin architecture, hydroxyl distribution, and crosslinking efficiency rather than only on total hydroxyl abundance [22,34,42,43].

**Table 8:** Two-Way ANOVA analysis of LF lap-shear strength for resin type, conditioning state, and interaction effects.

Source	df	SS	MS	F	p-value
Resin	2	1.256	0.628	2120.1	<0.0001
Condition	1	5.252	5.252	17733	<0.0001
Resin × Condition	2	0.104	0.052	175.3	<0.0001

**Table 9:** Tukey HSD post hoc grouping of LF lap-shear strength.

Resin	Condition	Mean ± SD (MPa)	Tukey Group*
CP	Dry	3.12 ± 0.01	a
ESD	Dry	2.85 ± 0.02	b
SB	Dry	2.34 ± 0.02	c
CP	Wet	1.92 ± 0.02	d
ESD	Wet	1.69 ± 0.01	e
SB	Wet	1.46 ± 0.01	f

**Note:** Means with different letters are significantly different according to Tukey HSD at p < 0.05.

The present study should be interpreted as a laboratory-scale comparative evaluation. Only three replicate lap-shear specimens were tested per treatment combination, and the PF benchmark was a laboratory control rather than a commercial adhesive. Lignin extraction yield was quantified, but residual carbohydrate content was not directly measured. Therefore, carbohydrate related effects on curing and adhesive performance were inferred directly from ash content, elemental composition, FTIR, and quantitative ^31^P NMR rather than confirmed by direct carbohydrate analysis. In addition, free formaldehyde was measured in liquid resin, but formaldehyde emission from finished panels was not evaluated. Accordingly, the findings support feedstock-dependent comparative performance under the tested conditions, but they should not be interpreted as proof of industrial equivalence or general applicability across wood substrates, panel types, or processing conditions.

### Scale-up feasibility and industrial consideration

Scale-up of LF resin production from local residues will require process and economic evaluation beyond laboratory synthesis. Important factors include lignin extraction yield, chemical consumption, NaOH and acid recovery, washing-water demand, drying energy, wastewater handling, feedstock seasonality, and transport cost [2,19]. CP lignin gave the highest extraction yield and best LF adhesive performance in this study, but chir pine needles would require organized collection and cleaning systems. SB is available from sugarcane-processing streams, but its lower lignin yield and weaker LF performance under the present conditions may require pretreatment or formulation optimization. Future pilot-scale work should evaluate mass balance, resin shelf life, panel-scale bonding, formaldehyde emissions, and comparison with commercial PF adhesives. Recent studies on natural fiber and biomass derived materials also show that feedstock selection, processing route, and application specific testing strongly influence final material performance [36,37].

## Conclusions

This study showed that unmodified alkaline lignins from chir pine needles, eucalyptus sawdust, and sugarcane bagasse can be used as the sole phenolic component in laboratory LF adhesive formulations. Lignin yield varied from 10.77 ± 0.31% for SB to 15.13 ± 0.21% for CP. Quantitative ^31^P NMR showed total OH + COOH values of 3.70 mmol g^-1^ for CP, 4.60 mmol g^-1^ for ESD, and 4.83 mmol g^-1^ for SB. These results show that total hydroxyl abundance alone did not determine adhesive performance. The LF resins showed alkaline pH, workable solids content, 3–4shear-thinning viscosity, and curing behavior suitable for laboratory hot pressing. Among the LF formulations, CP gave the highest dry and wet lap-shear strength, followed by ESD and SB. The laboratory PF control still showed higher dry and wet strengths than LF resins, and free formaldehyde was higher in the LF resins than in the PF control. Therefore, the present LF systems should be considered laboratory prototypes for lower-phenol adhesive development rather than direct commercial replacement. Future work should focus on residual carbohydrate quantification, reduction of free formaldehyde, panel emission testing, commercial PF benchmarking, moisture durability improvement, and pilot scale process evaluation.

## Supporting information

S1 Dataset Consolidated source data and statistical outputs for lignin-formaldehyde resin characterization and adhesive performance. (XLSX)

S2 Dataset FTIR source spectra and peak assignments. (XLSX)

S3 Dataset Primary 31P NMR data files. (XLSX)

S4 Dataset TGA source data and thermal summary. (XLSX)

S5 Dataset DSC curing summary data. (XLSX)

S6 Checklist Inclusivity in global research. Completed PLOS inclusivity in global research questionnaire for the revised submission.
(DOCX)

S7 Text 31P NMR acquisition parameters and integration metadata. (DOCX)
