# Supplementary material for: A lab-scale prototype development of Lignin-Formaldehyde (LF) resin: A bio-renewable adhesive for wood panel industries
Source: PLoS One. 2026 Jul 8;21(7):e0352893. doi: 10.1371/journal.pone.0352893 (PMC13345236; doi:10.1371/journal.pone.0352893)
Supplement: S7 Text — (DOCX) [file pone.0352893.s007.docx]

**S2 Text. ^31^P NMR acquisition parameters, integration regions, and hydroxyl quantification**

Manuscript: A lab-scale prototype development of Lignin-Formaldehyde (LF) resin: A bio-renewable adhesive for wood panel industries

Manuscript number: PONE-D-26-19567

# Purpose

This supporting text provides the NMR spectroscopy information requested during revision, including instrument details, magnetic-field class, nucleus measured, sample amount/concentration context, solvent, reference standard, temperature, acquisition parameters, integration regions, and sample-wise hydroxyl quantification. Primary acquisition and processed NMR data are provided separately as S3 Dataset.

# PLOS ^31^P NMR reporting checklist

| **Requested information** | **Provided information** |
| --- | --- |
| Instrument make/source and magnetic-field class | Bruker AVANCE III HD 600 MHz class NMR spectrometer; approximate ^31^P operating frequency 242.9 MHz; BBI multinuclear probe with Z-gradient. |
| Nucleus measured | ^31^P. |
| Sample concentration / sample amount | Approximately 40 mg oven-dried lignin dissolved in 0.5 mL dry anhydrous pyridine/CDCl_3_ (1.6:1, v/v), followed by reagents described below. |
| Solvent and solvent suppression | Dry anhydrous pyridine/CDCl_3_ (1.6:1, v/v). Solvent signal suppression was not used. |
| Reference standard | Cyclohexanol internal standard, referenced at 145.15 ppm. |
| Temperature | 298 K. |
| Acquisition parameters | 90 degree pulse angle, 10 s relaxation delay, inverse-gated proton decoupling, 256 scans, 20 ppm spectral window covering 152-132 ppm. |
| Processing parameters | 5 Hz line broadening, zero filling, phase correction, baseline correction, chemical-shift calibration, and region-wise integration. |
| Chemical shifts | Region-wise 31P integration ranges are reported in this file. The samples are derivatized technical lignins, not isolated small-molecule compounds; therefore, discrete multiplicity and coupling constants are not applicable. |
| Full integrated spectra and primary data | Provided in S3 Dataset, including acquisition/FID files, processing files, processed spectra, spectrum images/PDFs, and integration tables. |

# Sample preparation and derivatization

Approximately 40 mg of oven-dried lignin was used for each sample. The lignin was dried at 40ºC under vacuum for 24 h and stored over P_2_O_5_ before analysis. Each sample was dissolved in 0.5 mL of dry anhydrous pyridine/CDCl_3_ at a 1.6:1 volume ratio. Chromium(III) acetylacetonate [Cr(acac)_3_] was added at 0.05% (w/v) as relaxation agent. A 0.10 mL aliquot of cyclohexanol solution was used as the internal standard, followed by 0.05 mL of 2-chloro-4,4,5,5-tetramethyl-1,3,2-dioxaphospholane (TMDP) under dry nitrogen. The mixture was allowed to react for 15 min and then transferred to a sealed 5 mm NMR tube.

# Integration regions

| **Region** | **Chemical shift range (ppm)** | **Assignment purpose** |
| --- | --- | --- |
| Aliphatic OH | 149.1-145.4 | Aliphatic hydroxyl groups |
| Condensed-G OH | 144.6-143.3 | Condensed guaiacyl phenolic hydroxyls |
| Syringyl OH | 143.3-142.0 | Syringyl phenolic hydroxyls |
| Guaiacyl OH | 140.5-138.6 | Guaiacyl phenolic hydroxyls |
| p-Hydroxyphenyl OH | 138.5-137.3 | p-Hydroxyphenyl phenolic hydroxyls |
| Carboxylic OH | 135.9-134.0 | Carboxylic acid hydroxyl groups |

# Sample-wise hydroxyl quantification

| **Sample** | **Aliphatic OH** | **Condensed-G OH** | **S OH** | **G OH** | **H OH** | **COOH** | **Total phenolic OH** | **Total OH + COOH** |
| --- | --- | --- | --- | --- | --- | --- | --- | --- |
| CP lignin | 2.2 | 0.2 | 0.0 | 0.8 | 0.2 | 0.3 | 1.2 | 3.7 |
| ESD lignin | 2.15 | 0.29 | 1.03 | 0.43 | 0.1 | 0.6 | 1.85 | 4.6 |
| SB lignin | 1.21 | 0.58 | 0.82 | 1.39 | 0.31 | 0.53 | 3.09 | 4.83 |

# Reporting note

Because the samples are technical lignins derivatized for quantitative ^31^P NMR, the reported chemical-shift information is presented as functional-group integration regions. Multiplicity and coupling constants are not applicable to this quantitative lignin hydroxyl analysis.
