# Supplementary material for: A lab-scale prototype development of Lignin-Formaldehyde (LF) resin: A bio-renewable adhesive for wood panel industries
Source: PLoS One. 2026 Jul 8;21(7):e0352893. doi: 10.1371/journal.pone.0352893 (PMC13345236; doi:10.1371/journal.pone.0352893)
Supplement: S6 Checklist — Completed PLOS inclusivity in global research questionnaire for the revised submission. (DOCX) [file pone.0352893.s006.docx]

**Response to Reviewers comments**

PLOS ONE revised submission

Manuscript number: **PONE-D-26-19567**

Manuscript title: **A lab-scale prototype development of Lignin-Formaldehyde (LF) resin: A bio-renewable adhesive for wood panel industries**

Dear Respected Academic Editor and Reviewers,

We sincerely thank the Academic Editor and all reviewers for their careful assessment and constructive recommendations. We revised the manuscript and prepared the required supporting files. The revision clarifies the novelty, consolidates repetitive sections, adds lignin-yield and PF benchmark information, expands the discussion of lignin structure-reactivity-performance relationships, supplies additional source data, and addresses the PLOS-specific requirements on inclusivity and NMR reporting.

## Journal requirements

| **No.** | **Comment as provided by reviewer/editor** | **Response** |
| --- | --- | --- |
| 1 | Please ensure that your manuscript meets PLOS ONE's style requirements, including those for file naming. The PLOS ONE style templates can be found at  https://journals.plos.org/plosone/s/file?id=wjVg/PLOSOne_formatting_sample_main_body.pdf and https://journals.plos.org/plosone/s/file?id=ba62/PLOSOne_formatting_sample_title_authors_affiliations.pdf | The revised submission files have been organized using PLOS-compatible names. Main figures are supplied as separate TIFF files named Fig1.tif, Fig2.tif, Fig3.tif, and Fig4.tif. Supporting information files are named with S-number labels. The manuscript uses numbered references and consistent unit formatting. |
| 2 | Please include a complete copy of PLOS’s questionnaire on inclusivity in global research in your revised manuscript. Our policy for research in this area aims to improve transparency in the reporting of research performed outside of the researchers’ own country or community. The policy applies to researchers who have travelled to a different country to conduct research, research with Indigenous populations or their lands, and research on cultural artefacts. The questionnaire can also be requested at the journal’s discretion for any other submissions, even if these conditions are not met. Please find more information on the policy and a link to download a blank copy of the questionnaire here: https://journals.plos.org/plosone/s/best-practices-in-research-reporting. Please upload a completed version of your questionnaire as Supporting Information when you resubmit your manuscript. | A completed questionnaire has been prepared as S6 Checklist. 'Additional information regarding the ethical, cultural, and scientific considerations specific to inclusivity in global research is included in the Supporting Information (S6 Checklist).'  Location in revised manuscript: Supporting Information entry for S6 Checklist is listed on page 50, lines 1003-1004. The completed questionnaire is uploaded as S6 Checklist. |
| 3 | We note that this submission includes NMR spectroscopy data. We would recommend that you include the following information in your methods section or as Supporting Information files:  1) The make/source of the NMR instrument used in your study, as well as the magnetic field strength. For each individual experiment, please also list: the nucleus being measured; the sample concentration; the solvent in which the sample is dissolved and if solvent signal suppression was used; the reference standard and the temperature.  2) A list of the chemical shifts for all compounds characterised by NMR spectroscopy, specifying, where relevant: the chemical shift (δ), the multiplicity and the coupling constants (in Hz), for the appropriate nuclei used for assignment.  3) The full integrated NMR spectrum, clearly labelled with the compound name and chemical structure.  We also strongly encourage authors to provide primary NMR data files, in particular for new compounds which have not been characterised in the existing literature. Authors should provide the acquisition data, FID files and processing parameters for each experiment, clearly labelled with the compound name and identifier, as well as a structure file for each provided dataset. See our list of recommended repositories here: https://journals.plos.org/plosone/s/recommended-repositories. | The Methods section now reports key ^31^P NMR details, and a dedicated NMR supporting text has been prepared as S7 Text. Primary acquisition/processed NMR files, processed spectra, and integration tables are supplied in S3 Dataset. The S7 Text explains that the samples are derivatized technical lignins rather than discrete small molecules; therefore, region-wise ^31^P integration ranges are reported instead of small-molecule multiplicities/coupling constants. |
| 4 | If the reviewer comments include a recommendation to cite specific previously published works, please review and evaluate these publications to determine whether they are relevant and should be cited. There is no requirement to cite these works unless the editor has indicated otherwise. | Thank you for this instruction. We reviewed the recommended references and added the relevant recent studies to support the revised discussion on natural-material development and process optimization. The manuscript now cites these works in the discussion where optimization of LF adhesive formulation, processing conditions, and application-specific performance is discussed.  **Location in revised manuscript:** Lap-shear strength performance and moisture resistance section, Page 35, lines 700-703. |

## Reviewer #1

| **No.** | **Comment as provided by reviewer/editor** | **Response** |
| --- | --- | --- |
| 1 | The novelty of the study should be clarified more explicitly, particularly how this work differs from previously reported 100% lignin-substituted phenol-formaldehyde adhesives. | The Introduction was revised to state that complete phenol replacement is not claimed as the main novelty. The novelty is now framed as a comparative evaluation of unmodified alkaline lignins from Pakistani chir pine needles, eucalyptus sawdust, and sugarcane bagasse under matched extraction, resin synthesis, curing, and bonding conditions.  **Location in revised manuscript:** Introduction, pages 4-5, lines 82-90. |
| 2 | The lignin extraction yield was not reported, which limits assessment of process efficiency and industrial feasibility. | Lignin yield was added to the Results and Table 1. Yield values are CP 15.13 ± 0.21%, ESD 13.07 ± 0.38%, and SB 10.77 ± 0.31%. Raw yield replicates are included in S1 Dataset.  **Location in revised manuscript:** Results and Table 1, pages 17-18, lines 355-376; S1 Dataset entry, pages 49, lines 990-993. |
| 3 | Residual carbohydrate content in extracted lignin was not quantified, although it may strongly influence curing behavior and adhesive properties. | The Methods, Results, and limitations now state that residual carbohydrate content was not directly quantified. The discussion interprets carbohydrate effects cautiously using ash, elemental composition, FTIR, and ^31^P NMR, and future work is recommended to quantify residual carbohydrates directly.  **Location in revised manuscript:** Methods, page 8-9, lines 175-177; Results/discussion, pages 18, lines 385-388; limitations discussion, pages 37-38, lines 736-740; Conclusions, pages 39, lines 783-784. |
| 4 | The explanation relating hydroxyl content to adhesive performance requires stronger discussion supported by literature. | The Discussion now links lignin source, hydroxyl distribution, molecular size, reactive-site accessibility, curing behavior, crosslinking efficiency, and bond performance. It explains why SB had the highest total OH + COOH but did not give the highest lap-shear strength.  **Location in revised manuscript:** ^31^P NMR and hydroxyl-performance discussion, pages 24-25, lines 475-491; DSC/crosslinking discussion, pages 32, lines 624-635. |
| 5 | The FTIR interpretation contains several weak or uncommon band assignments that should be justified with references. | The FTIR section and Table 2 were revised. Weak bands in the 2000-1750 cm^-1^ region are now treated as overtone/combination-region signals and are not used as primary diagnostic evidence. The major interpretation relies on established O-H, aromatic skeletal, aryl-O, C-O, and syringyl/guaiacyl-related bands.  **Location in revised manuscript:** FTIR interpretation and Table 2 note, pages 19-21, lines 409-423; FTIR comparative interpretation, pages 22-23, lines 430-457. |
| 6 | The adhesive formulation includes wheat flour and alder bark additives, but their specific role in resin performance is insufficiently discussed. | The adhesive formulation section now explains that wheat flour was used as an extender/rheology modifier to improve spreadability and reduce excessive penetration, whereas alder bark material was used as a lignocellulosic filler to increase bond-line body and reduce resin loss into porous wood substrate.  **Location in revised manuscript:** adhesive formulation section, page 13, lines 264-276. |
| 7 | Units and symbols are inconsistently formatted throughout the manuscript, especially for temperature, viscosity, and molecular weight values. | Units and symbols were checked and standardized throughout the manuscript, including degrees C, Pa s, MPa, g mol^-1^, mmol g^-1^, and superscript/subscript formatting where required.  **Location in revised manuscript:** representative unit and symbol corrections are visible on page 2, lines 28-31; page 11, lines 220-225; page 26, lines 516-521; and pages 28-29, lines 560-573. |
| 8 | The manuscript language requires careful proofreading to correct grammatical issues and improve scientific clarity in several sections. | The manuscript was carefully edited for grammar, concision, and scientific clarity. Repetitive phrasing was reduced and long descriptive passages were consolidated.  **Location in revised manuscript:** proofreading and consolidation were applied throughout; representative revised text appears in the Abstract on pages 2-3, lines 21-52, and the Introduction on pages 4-6, lines 66-127. |
| 9 | The discussion section (Statistical analysis, ANOVA) is mostly descriptive and requires deeper scientific interpretation supported by relevant mechanisms and literature.  Analysis of erosion wear behavior of LD sludge filled polypropylene composites using RSM.  Analysis of sliding wear behavior of LD sludge filled epoxy composites using response surface methodology. | The ANOVA/Tukey discussion was expanded to interpret significant resin, conditioning, and interaction effects in terms of feedstock-dependent cured-network development and moisture resistance.  **Location in revised manuscript:** Statistical analysis methods, page 16, lines 344-350; expanded ANOVA/Tukey interpretation, pages 36, lines 706-727; S1 Dataset entry, pages 49, lines 990-993 and 1018-1019. |
| 10 | Figure captions are inconsistent in formatting style and should include experimental conditions for better interpretation. | Figure captions were revised to include sample identities and key conditions, such as FTIR scan range/resolution, ^31^P NMR derivatization and acquisition details, TGA temperature range/heating rate/nitrogen flow, and DSC heating conditions.  **Location in revised manuscript:** revised figure captions are shown for FTIR on page 21, lines 424-426; ^31^P NMR on page 24, lines 468-472; TGA/DTG on page 27, lines 548-551; and DSC on page 30, lines 595-598. |
| 11 | In conclusion, be more specific in applications too. Add technical facts to the conclusions to improve them. | The Conclusions section now reports key technical values and states that the LF systems are laboratory prototypes for lower-phenol adhesive development rather than direct commercial replacements. Future work on formaldehyde emissions, commercial PF benchmarking, durability, and pilot-scale feasibility is also specified.  **Location in revised manuscript:** Conclusions, pages 38-40, lines 760-786. |

## Reviewer #2

**General reviewer statement:** The manuscript addresses the development of lignin–formaldehyde (LF) resins using lignins from different biomass sources and evaluates their performance as wood adhesives. While the topic is relevant in the context of sustainable materials, the manuscript suffers from limited novelty, structural weaknesses, and insufficient critical discussion, which currently reduces its contribution to the field.

| **No.** | **Comment as provided by reviewer/editor** | **Response** |
| --- | --- | --- |
| 1 | Comparing lignins from different biomass sources and their influence on phenol-formaldehyde resin performance is not sufficiently novel. Several prior studies have already demonstrated this topic and yet there was not sufficient review presented in the introduction. Please strengthen the novelty by clearly defining what is genuinely new. | The Introduction was revised to distinguish the study from earlier 100% lignin replacement work. The revised novelty is the matched comparison of three unmodified local Pakistani biomass residues across lignin structure, resin processing, curing, and adhesive performance.  **Location in revised manuscript:** Introduction, pages 4-5, lines 82-90. |
| 2 | The manuscript is overly long and repetitive, particularly in:  a. the Abstract that is too dense with numerical results rather than highlighting key findings/trends and overall conclusions  b. Lignin extraction procedures (repeated almost identically for each feedstock)  c. Characterization descriptions (FTIR, TGA, NMR)  d. Result presentation (each lignin and resin discussed separately with similar narrative structure)  I recommend consolidating methods into generic protocols with feedstock-specific deviations summarized in tables, results into comparative sections (CP vs ESD vs SB) instead of sequential descriptions, merge figures where possible rather than separate figures per lignin. | The Abstract was revised to emphasize the main trends and interpretation rather than listing excessive numerical details.  The Methods were consolidated into a single generic alkali extraction protocol, with all feedstocks treated under the same conditions.  The FTIR, TGA, NMR, DSC, and lap-shear sections were reorganized into comparative subsections, reducing repeated sample-by-sample narrative.  Figures were reorganized as multi-panel figures. The final figure set includes FTIR, ^31^P NMR, TGA/DTG, and DSC figures, supplied separately as PLOS-ready TIFF files.  **Location in revised manuscript:** revised Abstract, pages 2-3, lines 21-52; consolidated extraction method, pages 7, lines 148-153 and 172-176; comparative Results sections, pages 17-19, lines 355-403; merged figure captions, page 21, lines 424-426, page 24, lines 468-472, page 27, lines 548-551, and page 30, lines 595-598. |
| 3 | The discussion remains largely descriptive rather than analytical. Key observations are reported but not deeply interpreted. Strengthen discussion by  a. linking lignin structure → reactivity → crosslink density → adhesive performance  b. comparing findings critically with literature (agreement vs deviation)  c. providing mechanistic explanations (e.g., steric hindrance in the different phenolic groups, accessibility of reactive sites, role of molecular weight) | The Discussion now explicitly connects lignin source and hydroxyl distribution to methylolation, condensation, crosslink-network development, Tg, gel time, and dry/wet lap-shear strength.  The Discussion was expanded with relevant literature on lignin phenolic adhesives, hydroxyl functionality, molecular weight, and adhesive performance.  Mechanistic interpretation was added, particularly explaining why total hydroxyl abundance alone did not control adhesive performance.  **Location in revised manuscript:** mechanistic structure-reactivity-performance discussion, pages 24-25, lines 475-491; pages 30-32, lines 601-635; pages 33-34, lines 649-684; and statistical interpretation, pages 36, lines 707-727. |
| 4 | The methodology mentions different levels of lignin substitution (0%, 20%, 50%, 80%, 100%), but only one resin per lignin type (apparently at full substitution) is presented in the results, and it´s not clear if this is from a 100% substitution? If it is indeed 100%, the reported viscosity (~0.3 Pa·s at ~50% solids) also seems unusually low, likely due to the very high shear rate (1000 s⁻¹). Although such shear conditions may be relevant for industrial processing, they could mask subtle but potentially important differences arising from formulation changes or lignin type variations especially true for shear thinning phenolic resins. Measurements at lower or multiple shear rates (e.g., 50–100 s⁻¹) would be more appropriate for comparison. | The Methods now clearly states that only complete phenol-replacement LF resins are reported and earlier lower-substitution trials are not included in the dataset. New multi-shear-rate viscosity data from 1 to 1000 s^-1^ were added, showing shear-thinning behavior. These values are included in S1 Dataset.  **Location in revised manuscript:** complete phenol-replacement clarification, page 12, lines 247-259; multi-shear-rate viscosity methods, page 13-14, lines 285-292; viscosity results and Table 5, pages 28-29, lines 560-573; S1 Dataset entry, pages 49, lines 990-993. |

## Reviewer #3

**General reviewer statement:** The manuscript presents an interesting comparative study on lignin-formaldehyde bioadhesives synthesized from locally available biomass resources. The work is relevant to sustainable adhesive development and circular bioeconomy research. The manuscript is generally well-structured and supported by multiple characterization techniques including FTIR, NMR, TGA, DSC, and lap-shear testing. However, several scientific, methodological, and presentation-related issues should be addressed before the manuscript can be considered for publication.

| **No.** | **Comment as provided by reviewer/editor** | **Response** |
| --- | --- | --- |
| 1 | Include at least one commercial phenol-formaldehyde adhesive as a control for comparative bonding performance. | A laboratory PF control was added as a benchmark for resin properties and lap-shear performance. Because no commercial PF dataset was available, the revised manuscript explicitly states that this control is a laboratory benchmark and not a commercial adhesive.  **Location in revised manuscript:** laboratory PF control method clarification, pages 12, lines 259-263; PF comparison in resin properties, page 28, lines 553-558; lap-shear benchmark results, pages 33-35, lines 658-695; limitations clarification, page 37-38, lines 733-745. |
| 2 | Since the adhesive system is formaldehyde-based, formaldehyde emission analysis is critical for environmental and industrial applicability. The manuscript lacks any emission or toxicity evaluation. | Free formaldehyde in liquid resin was added and reported for the PF control and all LF resins. The manuscript clearly states that these values are not finished-panel emissions and identifies panel formaldehyde emission testing as a necessary future step.  **Location in revised manuscript:** free formaldehyde method, pages 14-15, lines 304-308; resin-level formaldehyde results, page 30, lines 584-591; limitation on finished-panel emissions, page 38, lines 740-742; Conclusions, pages 39-40, lines 770-772. |
| 3 | Add a section discussing scale-up feasibility, economic considerations, and industrial implementation challenges. | A new section on scale-up feasibility and industrial considerations was added. It discusses extraction yield, chemical consumption, recovery, washing-water demand, drying energy, wastewater handling, feedstock seasonality, transport, resin shelf life, panel-scale bonding, emissions, and commercial benchmarking.  **Location in revised manuscript:** Scale-up feasibility and industrial consideration section, page 38, lines 746-758; future work in Conclusions, pages 39-40, lines 783-786. |
| 4 | The manuscript claims novelty based on comparison of Pakistani biomass feedstocks. However, complete phenol substitution using lignin has already been reported in earlier studies. Clarify the specific scientific novelty and distinguish this work more clearly from prior lignin-PF adhesive studies. | The Introduction and Conclusions now clarify that the novelty is not complete phenol substitution itself, but a controlled feedstock-dependent comparison of locally available Pakistani biomass lignins under identical LF resin preparation and testing conditions.  **Location in revised manuscript:** Introduction, pages 4-5, lines 82-90; Conclusions, pages 39-40, lines 760-786. |
| 5 | There are inconsistencies in formatting: “1000 C” instead of “1000°C” Mixed spacing around units and symbols  Inconsistent use of superscripts/subscripts. Carefully revise unit formatting according to journal style. | Unit formatting was standardized throughout the manuscript and tables.  **Location in revised manuscript:** representative formatting corrections are visible on page 2, lines 31-34; page 11, lines 220-225; page 26, lines 516-521; and pages 30, lines 601-604. |
| 6 | Discuss moisture resistance limitations and possible methods for improving wet durability. | The lap-shear discussion now includes wet/dry retention, wet-strength limitations, and improvement routes such as lignin activation, formaldehyde-to-lignin ratio adjustment, NaOH optimization, filler loading, pressing schedule optimization, and bio-based crosslinking strategies.  **Location in revised manuscript:** moisture-resistance discussion, pages 33-35, lines 648-705. |
| 7 | The role of wheat flour and alder bark modal in the adhesive formulation is not adequately explained. | The adhesive formulation section now explains the functional roles of wheat flour and alder bark material, and notes that their loading was kept constant across all formulations.  **Location in revised manuscript:** adhesive formulation section, page 13, lines 264-276. |
| 8 | The authors have carried out the present study using natural biomass-derived materials and optimization of lignin-based adhesive formulations; however, the manuscript lacks sufficient citation of recent and relevant literature related to natural-material-based material development and optimization studies. The authors are encouraged to include and discuss recent published works in this area. Some recommended references relevant to the present study are as follows: https://doi.org/10.1007/s40964-025-01478-7  https://doi.org/10.1016/j.envres.2022.115021  https://doi.org/10.1177/09544089241253973  https://doi.org/10.1007/s11665-024-10122-4 | Thank you for this suggestion. Relevant recent literature on natural-material-based development and optimization studies has been added and discussed in the revised manuscript. The discussion now highlights that LF adhesive performance depends on formulation variables, processing conditions, and application-specific testing, and that future development should include structured optimization approaches. **Location in revised manuscript:** Lap-shear strength performance and moisture resistance section, Page 35, lines 700-703. |

## Supporting files prepared with the revision

S1 Dataset. Consolidated source data and reported summary values for lignin-formaldehyde resin characterization and adhesive performance.

S2 Dataset. FTIR source spectra, raw data, and peak assignments for CP, ESD, and SB lignins.

S3 Dataset. 31P NMR source spectra, primary acquisition/processed files, and hydroxyl-content data for CP, ESD, and SB lignins.

S4 Dataset. TGA source data and reported thermal summary for CP, ESD, and SB lignins.

S5 Dataset. DSC reported thermal summary for CP, ESD, and SB lignin-formaldehyde resins.

S6 Checklist. Inclusivity in global research.

S7 Text. 31P NMR acquisition parameters, integration regions, and hydroxyl quantification.

Fig1.tif-Fig4.tif. PLOS-ready figure files.

## Remaining study limitations stated transparently

The revised manuscript explicitly states that residual carbohydrate content, finished-panel formaldehyde emissions, commercial PF benchmarking, and broader panel-scale validation remain future requirements. These are limitations of the available dataset rather than omissions in the revised reporting.

Sincerely,
**Dr. Majid Hussain**
Associate Professor/Lead of Industrial and Wood Technology Lab

Department of Forestry & Wildlife Management,

University of Haripur, Pakistan
majid@uoh.edu.pk | (+92) 3088144348
