# Supplementary material for: A lab-scale prototype development of Lignin-Formaldehyde (LF) resin: A bio-renewable adhesive for wood panel industries
Source: PLoS One. 2026 Jul 8;21(7):e0352893. doi: 10.1371/journal.pone.0352893 (PMC13345236; doi:10.1371/journal.pone.0352893)
Supplement: Peer review history

# Peer review: Decision Letter 0

**DOI:** 10.1371/journal.pone.0352893.r001

**Authors:** Din Bandhu

19 May 2026

PONE-D-26-19567

A lab-scale prototype development of Lignin-Formaldehyde (LF) resin: A bio-renewable adhesive for wood panel industries

PLOS One

Dear Dr. Hussain,

Thank you for submitting your manuscript to PLOS ONE. After careful consideration, we feel that it has merit but does not fully meet PLOS ONE’s publication criteria as it currently stands. Therefore, we invite you to submit a revised version of the manuscript that addresses the points raised during the review process.

Please submit your revised manuscript by Jul 03 2026 11:59PM. If you will need more time than this to complete your revisions, please reply to this message or contact the journal office at plosone@plos.org. When you're ready to submit your revision, log on to https://www.editorialmanager.com/pone/ and select the 'Submissions Needing Revision' folder to locate your manuscript file.

Please include the following items when submitting your revised manuscript:

- A letter that responds to each point raised by the academic editor and reviewer(s). You should upload this letter as a separate file labeled 'Response to Reviewers'.
- A marked-up copy of your manuscript that highlights changes made to the original version. You should upload this as a separate file labeled 'Revised Manuscript with Track Changes'.
- An unmarked version of your revised paper without tracked changes. You should upload this as a separate file labeled 'Manuscript'.

If you would like to make changes to your financial disclosure, please include your updated statement in your cover letter. Guidelines for resubmitting your figure files are available below the reviewer comments at the end of this letter.

If applicable, we recommend that you deposit your laboratory protocols in protocols.io to enhance the reproducibility of your results. Protocols.io assigns your protocol its own identifier (DOI) so that it can be cited independently in the future. For instructions see: https://journals.plos.org/plosone/s/submission-guidelines#loc-laboratory-protocols. Additionally, PLOS ONE offers an option for publishing peer-reviewed Lab Protocol articles, which describe protocols hosted on protocols.io. Read more information on sharing protocols at https://plos.org/protocols?utm_medium=editorial-email&utm_source=authorletters&utm_campaign=protocols.

As the corresponding author, your ORCID iD is verified in the submission system and will appear in the published article. PLOS supports the use of ORCID, and we encourage all coauthors to register for an ORCID iD and use it as well. Please encourage your coauthors to verify their ORCID iD within the submission system before final acceptance, as unverified ORCID iDs will not appear in the published article. *Only*  the individual author can complete the verification step; PLOS staff *cannot*  verify ORCID iDs on behalf of authors.

We look forward to receiving your revised manuscript.

Kind regards,

Din Bandhu, Ph.D.

Academic Editor

PLOS One

Journal Requirements:

When submitting your revision, we need you to address these additional requirements.

1. Please ensure that your manuscript meets PLOS ONE's style requirements, including those for file naming. The PLOS ONE style templates can be found at

https://journals.plos.org/plosone/s/file?id=wjVg/PLOSOne_formatting_sample_main_body.pdf and https://journals.plos.org/plosone/s/file?id=ba62/PLOSOne_formatting_sample_title_authors_affiliations.pdf

2. Please include a complete copy of PLOS’s questionnaire on inclusivity in global research in your revised manuscript. Our policy for research in this area aims to improve transparency in the reporting of research performed outside of the researchers’ own country or community. The policy applies to researchers who have travelled to a different country to conduct research, research with Indigenous populations or their lands, and research on cultural artefacts. The questionnaire can also be requested at the journal’s discretion for any other submissions, even if these conditions are not met.  Please find more information on the policy and a link to download a blank copy of the questionnaire here: https://journals.plos.org/plosone/s/best-practices-in-research-reporting. Please upload a completed version of your questionnaire as Supporting Information when you resubmit your manuscript.

3. We note that this submission includes NMR spectroscopy data. We would recommend that you include the following information in your methods section or as Supporting Information files:

1) The make/source of the NMR instrument used in your study, as well as the magnetic field strength. For each individual experiment, please also list: the nucleus being measured; the sample concentration; the solvent in which the sample is dissolved and if solvent signal suppression was used; the reference standard and the temperature.

2) A list of the chemical shifts for all compounds characterised by NMR spectroscopy, specifying, where relevant: the chemical shift (δ), the multiplicity and the coupling constants (in Hz), for the appropriate nuclei used for assignment.

3)The full integrated NMR spectrum, clearly labelled with the compound name and chemical structure.

We also strongly encourage authors to provide primary NMR data files, in particular for new compounds which have not been characterised in the existing literature. Authors should provide the acquisition data, FID files and processing parameters for each experiment, clearly labelled with the compound name and identifier, as well as a structure file for each provided dataset. See our list of recommended repositories here: https://journals.plos.org/plosone/s/recommended-repositories.

4. If the reviewer comments include a recommendation to cite specific previously published works, please review and evaluate these publications to determine whether they are relevant and should be cited. There is no requirement to cite these works unless the editor has indicated otherwise.

[Note: HTML markup is below. Please do not edit.]

Reviewers' comments:

Reviewer's Responses to Questions

**Comments to the Author**

1. Is the manuscript technically sound, and do the data support the conclusions?

The manuscript must describe a technically sound piece of scientific research with data that supports the conclusions. Experiments must have been conducted rigorously, with appropriate controls, replication, and sample sizes. The conclusions must be drawn appropriately based on the data presented.

Reviewer #1: Yes

Reviewer #2: Partly

Reviewer #3: Yes

**********

2. Has the statistical analysis been performed appropriately and rigorously?

Reviewer #1: Yes

Reviewer #2: Yes

Reviewer #3: Yes

**********

3. Have the authors made all data underlying the findings in their manuscript fully available?

The PLOS Data policy requires authors to make all data underlying the findings described in their manuscript fully available without restriction, with rare exception (please refer to the Data Availability Statement in the manuscript PDF file). The data should be provided as part of the manuscript or its supporting information, or deposited to a public repository. For example, in addition to summary statistics, the data points behind means, medians and variance measures should be available. If there are restrictions on publicly sharing data—e.g. participant privacy or use of data from a third party—those must be specified.

Reviewer #1: Yes

Reviewer #2: Yes

Reviewer #3: Yes

**********

4. Is the manuscript presented in an intelligible fashion and written in standard English?

PLOS ONE does not copyedit accepted manuscripts, so the language in submitted articles must be clear, correct, and unambiguous. Any typographical or grammatical errors should be corrected at revision, so please note any specific errors here.

Reviewer #1: Yes

Reviewer #2: No

Reviewer #3: Yes

**********

5. Review Comments to the Author

Please use the space provided to explain your answers to the questions above. You may also include additional comments for the author, including concerns about dual publication, research ethics, or publication ethics. (Please upload your review as an attachment if it exceeds 20,000 characters)

Reviewer #1: 1. The novelty of the study should be clarified more explicitly, particularly how this work differs from previously reported 100% lignin-substituted phenol-formaldehyde adhesives.

2. The lignin extraction yield was not reported, which limits assessment of process efficiency and industrial feasibility.

3. Residual carbohydrate content in extracted lignin was not quantified, although it may strongly influence curing behavior and adhesive properties.

4. The explanation relating hydroxyl content to adhesive performance requires stronger discussion supported by literature.

5. The FTIR interpretation contains several weak or uncommon band assignments that should be justified with references.

6. The adhesive formulation includes wheat flour and alder bark additives, but their specific role in resin performance is insufficiently discussed.

7. Units and symbols are inconsistently formatted throughout the manuscript, especially for temperature, viscosity, and molecular weight values.

8. The manuscript language requires careful proofreading to correct grammatical issues and improve scientific clarity in several sections.

9. The discussion section (Statistical analysis, ANOVA) is mostly descriptive and requires deeper scientific interpretation supported by relevant mechanisms and literature.

Analysis of erosion wear behavior of LD sludge filled polypropylene composites using RSM.

Analysis of sliding wear behavior of LD sludge filled epoxy composites using response surface methodology.

10. Figure captions are inconsistent in formatting style and should include experimental conditions for better interpretation.

11. In conclusion, be more specific in applications too. Add technical facts to the conclusions to improve them.

Reviewer #2: The manuscript addresses the development of lignin–formaldehyde (LF) resins using lignins from different biomass sources and evaluates their performance as wood adhesives. While the topic is relevant in the context of sustainable materials, the manuscript suffers from limited novelty, structural weaknesses, and insufficient critical discussion, which currently reduces its contribution to the field.

Major Comments

1. Comparing lignins from different biomass sources and their influence on phenol-formaldehyde resin performance is not sufficiently novel. Several prior studies have already demonstrated this topic and yet there was not sufficient review presented in the introduction. Please strengthen the novelty by clearly defining what is genuinely new.

2. The manuscript is overly long and repetitive, particularly in:

a. the Abstract that is too dense with numerical results rather than highlighting key findings/trends and overall conclusions

b. Lignin extraction procedures (repeated almost identically for each feedstock)

c. Characterization descriptions (FTIR, TGA, NMR)

d. Result presentation (each lignin and resin discussed separately with similar narrative structure)

I recommend consolidating methods into generic protocols with feedstock-specific deviations summarized in tables, results into comparative sections (CP vs ESD vs SB) instead of sequential descriptions, merge figures where possible rather than separate figures per lignin.

3. The discussion remains largely descriptive rather than analytical. Key observations are reported but not deeply interpreted. Strengthen discussion by

a. linking lignin structure → reactivity → crosslink density → adhesive performance

b. comparing findings critically with literature (agreement vs deviation)

c. providing mechanistic explanations (e.g., steric hindrance in the different phenolic groups, accessibility of reactive sites, role of molecular weight)

4. The methodology mentions different levels of lignin substitution (0%, 20%, 50%, 80%, 100%), but only one resin per lignin type (apparently at full substitution) is presented in the results, and it´s not clear if this is from a 100% substitution? If it is indeed 100%, the reported viscosity (~0.3 Pa·s at ~50% solids) also seems unusually low, likely due to the very high shear rate (1000 s⁻¹). Although such shear conditions may be relevant for industrial processing, they could mask subtle but potentially important differences arising from formulation changes or lignin type variations especially true for shear thinning phenolic resins. Measurements at lower or multiple shear rates (e.g., 50–100 s⁻¹) would be more appropriate for comparison.

Reviewer #3: The manuscript presents an interesting comparative study on lignin-formaldehyde bioadhesives synthesized from locally available biomass resources. The work is relevant to sustainable adhesive development and circular bioeconomy research. The manuscript is generally well-structured and supported by multiple characterization techniques including FTIR, NMR, TGA, DSC, and lap-shear testing. However, several scientific, methodological, and presentation-related issues should be addressed before the manuscript can be considered for publication.

1. Include at least one commercial phenol-formaldehyde adhesive as a control for comparative bonding performance.

2. Since the adhesive system is formaldehyde-based, formaldehyde emission analysis is critical for environmental and industrial applicability. The manuscript lacks any emission or toxicity evaluation.

3. Add a section discussing scale-up feasibility, economic considerations, and industrial implementation challenges.

4. The manuscript claims novelty based on comparison of Pakistani biomass feedstocks. However, complete phenol substitution using lignin has already been reported in earlier studies. Clarify the specific scientific novelty and distinguish this work more clearly from prior lignin-PF adhesive studies.

5. There are inconsistencies in formatting: “1000 C” instead of “1000°C” Mixed spacing around units and symbols

Inconsistent use of superscripts/subscripts. Carefully revise unit formatting according to journal style.

6. Discuss moisture resistance limitations and possible methods for improving wet durability.

7. The role of wheat flour and alder bark modal in the adhesive formulation is not adequately explained.

8.The authors have carried out the present study using natural biomass-derived materials and optimization of lignin-based adhesive formulations; however, the manuscript lacks sufficient citation of recent and relevant literature related to natural-material-based material development and optimization studies. The authors are encouraged to include and discuss recent published works in this area. Some recommended references relevant to the present study are as follows: https://doi.org/10.1007/s40964-025-01478-7

https://doi.org/10.1016/j.envres.2022.115021

https://doi.org/10.1177/09544089241253973

https://doi.org/10.1007/s11665-024-10122-4

**********

6. PLOS authors have the option to publish the peer review history of their article (what does this mean?). If published, this will include your full peer review and any attached files.

If you choose “no”, your identity will remain anonymous but your review may still be made public.

**Do you want your identity to be public for this peer review?**  For information about this choice, including consent withdrawal, please see our Privacy Policy.

Reviewer #1: No

Reviewer #2: No

Reviewer #3: No

**********

[NOTE: If reviewer comments were submitted as an attachment file, they will be attached to this email and accessible via the submission site. Please log into your account, locate the manuscript record, and check for the action link "View Attachments". If this link does not appear, there are no attachment files.]

To ensure your figures meet our technical requirements, please review our figure guidelines: https://journals.plos.org/plosone/s/figures

You may also use PLOS’s free figure tool, NAAS, to help you prepare publication quality figures: https://journals.plos.org/plosone/s/figures#loc-tools-for-figure-preparation.

NAAS will assess whether your figures meet our technical requirements by comparing each figure against our figure specifications.

# Author comment: Author response to Decision Letter 1

**DOI:** 10.1371/journal.pone.0352893.r002

6 Jun 2026

Response to Reviewers comments

PLOS ONE revised submission

Manuscript number: PONE-D-26-19567

Manuscript title: A lab-scale prototype development of Lignin-Formaldehyde (LF) resin: A bio-renewable adhesive for wood panel industries

Dear Respected Academic Editor and Reviewers,

We sincerely thank the Academic Editor and all reviewers for their careful assessment and constructive recommendations. We revised the manuscript and prepared the required supporting files. The revision clarifies the novelty, consolidates repetitive sections, adds lignin-yield and PF benchmark information, expands the discussion of lignin structure-reactivity-performance relationships, supplies additional source data, and addresses the PLOS-specific requirements on inclusivity and NMR reporting.

Journal requirements

No. Comment as provided by reviewer/editor Response

1 Please ensure that your manuscript meets PLOS ONE's style requirements, including those for file naming. The PLOS ONE style templates can be found at

https://journals.plos.org/plosone/s/file?id=wjVg/PLOSOne_formatting_sample_main_body.pdf and https://journals.plos.org/plosone/s/file?id=ba62/PLOSOne_formatting_sample_title_authors_affiliations.pdf The revised submission files have been organized using PLOS-compatible names. Main figures are supplied as separate TIFF files named Fig1.tif, Fig2.tif, Fig3.tif, and Fig4.tif. Supporting information files are named with S-number labels. The manuscript uses numbered references and consistent unit formatting.

2 Please include a complete copy of PLOS’s questionnaire on inclusivity in global research in your revised manuscript. Our policy for research in this area aims to improve transparency in the reporting of research performed outside of the researchers’ own country or community. The policy applies to researchers who have travelled to a different country to conduct research, research with Indigenous populations or their lands, and research on cultural artefacts. The questionnaire can also be requested at the journal’s discretion for any other submissions, even if these conditions are not met. Please find more information on the policy and a link to download a blank copy of the questionnaire here: https://journals.plos.org/plosone/s/best-practices-in-research-reporting. Please upload a completed version of your questionnaire as Supporting Information when you resubmit your manuscript. A completed questionnaire has been prepared as S6 Checklist. 'Additional information regarding the ethical, cultural, and scientific considerations specific to inclusivity in global research is included in the Supporting Information (S6 Checklist).'

Location in revised manuscript: Supporting Information entry for S6 Checklist is listed on page 50, lines 1003-1004. The completed questionnaire is uploaded as S6 Checklist.

3 We note that this submission includes NMR spectroscopy data. We would recommend that you include the following information in your methods section or as Supporting Information files:

1) The make/source of the NMR instrument used in your study, as well as the magnetic field strength. For each individual experiment, please also list: the nucleus being measured; the sample concentration; the solvent in which the sample is dissolved and if solvent signal suppression was used; the reference standard and the temperature.

2) A list of the chemical shifts for all compounds characterised by NMR spectroscopy, specifying, where relevant: the chemical shift (δ), the multiplicity and the coupling constants (in Hz), for the appropriate nuclei used for assignment.

3) The full integrated NMR spectrum, clearly labelled with the compound name and chemical structure.

We also strongly encourage authors to provide primary NMR data files, in particular for new compounds which have not been characterised in the existing literature. Authors should provide the acquisition data, FID files and processing parameters for each experiment, clearly labelled with the compound name and identifier, as well as a structure file for each provided dataset. See our list of recommended repositories here: https://journals.plos.org/plosone/s/recommended-repositories. The Methods section now reports key 31P NMR details, and a dedicated NMR supporting text has been prepared as S7 Text. Primary acquisition/processed NMR files, processed spectra, and integration tables are supplied in S3 Dataset. The S7 Text explains that the samples are derivatized technical lignins rather than discrete small molecules; therefore, region-wise 31P integration ranges are reported instead of small-molecule multiplicities/coupling constants.

4 If the reviewer comments include a recommendation to cite specific previously published works, please review and evaluate these publications to determine whether they are relevant and should be cited. There is no requirement to cite these works unless the editor has indicated otherwise. Thank you for this instruction. We reviewed the recommended references and added the relevant recent studies to support the revised discussion on natural-material development and process optimization. The manuscript now cites these works in the discussion where optimization of LF adhesive formulation, processing conditions, and application-specific performance is discussed.

Location in revised manuscript: Lap-shear strength performance and moisture resistance section, Page 35, lines 700-703.

Reviewer #1

No. Comment as provided by reviewer/editor Response

1 The novelty of the study should be clarified more explicitly, particularly how this work differs from previously reported 100% lignin-substituted phenol-formaldehyde adhesives. The Introduction was revised to state that complete phenol replacement is not claimed as the main novelty. The novelty is now framed as a comparative evaluation of unmodified alkaline lignins from Pakistani chir pine needles, eucalyptus sawdust, and sugarcane bagasse under matched extraction, resin synthesis, curing, and bonding conditions.

Location in revised manuscript: Introduction, pages 4-5, lines 82-90.

2 The lignin extraction yield was not reported, which limits assessment of process efficiency and industrial feasibility. Lignin yield was added to the Results and Table 1. Yield values are CP 15.13 ± 0.21%, ESD 13.07 ± 0.38%, and SB 10.77 ± 0.31%. Raw yield replicates are included in S1 Dataset.

Location in revised manuscript: Results and Table 1, pages 17-18, lines 355-376; S1 Dataset entry, pages 49, lines 990-993.

3 Residual carbohydrate content in extracted lignin was not quantified, although it may strongly influence curing behavior and adhesive properties. The Methods, Results, and limitations now state that residual carbohydrate content was not directly quantified. The discussion interprets carbohydrate effects cautiously using ash, elemental composition, FTIR, and 31P NMR, and future work is recommended to quantify residual carbohydrates directly.

Location in revised manuscript: Methods, page 8-9, lines 175-177; Results/discussion, pages 18, lines 385-388; limitations discussion, pages 37-38, lines 736-740; Conclusions, pages 39, lines 783-784.

4 The explanation relating hydroxyl content to adhesive performance requires stronger discussion supported by literature. The Discussion now links lignin source, hydroxyl distribution, molecular size, reactive-site accessibility, curing behavior, crosslinking efficiency, and bond performance. It explains why SB had the highest total OH + COOH but did not give the highest lap-shear strength.

Location in revised manuscript: 31P NMR and hydroxyl-performance discussion, pages 24-25, lines 475-491; DSC/crosslinking discussion, pages 32, lines 624-635.

5 The FTIR interpretation contains several weak or uncommon band assignments that should be justified with references. The FTIR section and Table 2 were revised. Weak bands in the 2000-1750 cm-1 region are now treated as overtone/combination-region signals and are not used as primary diagnostic evidence. The major interpretation relies on established O-H, aromatic skeletal, aryl-O, C-O, and syringyl/guaiacyl-related bands.

Location in revised manuscript: FTIR interpretation and Table 2 note, pages 19-21, lines 409-423; FTIR comparative interpretation, pages 22-23, lines 430-457.

6 The adhesive formulation includes wheat flour and alder bark additives, but their specific role in resin performance is insufficiently discussed. The adhesive formulation section now explains that wheat flour was used as an extender/rheology modifier to improve spreadability and reduce excessive penetration, whereas alder bark material was used as a lignocellulosic filler to increase bond-line body and reduce resin loss into porous wood substrate.

Location in revised manuscript: adhesive formulation section, page 13, lines 264-276.

7 Units and symbols are inconsistently formatted throughout the manuscript, especially for temperature, viscosity, and molecular weight values. Units and symbols were checked and standardized throughout the manuscript, including degrees C, Pa s, MPa, g mol-1, mmol g-1, and superscript/subscript formatting where required.

Location in revised manuscript: representative unit and symbol corrections are visible on page 2, lines 28-31; page 11, lines 220-225; page 26, lines 516-521; and pages 28-29, lines 560-573.

8 The manuscript language requires careful proofreading to correct grammatical issues and improve scientific clarity in several sections. The manuscript was carefully edited for grammar, concision, and scientific clarity. Repetitive phrasing was reduced and long descriptive passages were consolidated.

Location in revised manuscript: proofreading and consolidation were applied throughout; representative revised text appears in the Abstract on pages 2-3, lines 21-52, and the Introduction on pages 4-6, lines 66-127.

9 The discussion section (Statistical analysis, ANOVA) is mostly descriptive and requires deeper scientific interpretation supported by relevant mechanisms and literature.

Analysis of erosion wear behavior of LD sludge filled polypropylene composites using RSM.

Analysis of sliding wear behavior of LD sludge filled epoxy composites using response surface methodology. The ANOVA/Tukey discussion was expanded to interpret significant resin, conditioning, and interaction effects in terms of feedstock-dependent cured-network development and moisture resistance.

Location in revised manuscript: Statistical analysis methods, page 16, lines 344-350; expanded ANOVA/Tukey interpretation, pages 36, lines 706-727; S1 Dataset entry, pages 49, lines 990-993 and 1018-1019.

10 Figure captions are inconsistent in formatting style and should include experimental conditions for better interpretation. Figure captions were revised to include sample identities and key conditions, such as FTIR scan range/resolution, 31P NMR derivatization and acquisition details, TGA temperature range/heating rate/nitrogen flow, and DSC heating conditions.

Location in revised manuscript: revised figure captions are shown for FTIR on page 21, lines 424-426; 31P NMR on page 24, lines 468-472; TGA/DTG on page 27, lines 548-551; and DSC on page 30, lines 595-598.

11 In conclusion, be more specific in applications too. Add technical facts to the conclusions to improve them. The Conclusions section now reports key technical values and states that the LF systems are laboratory prototypes for lower-phenol adhesive development rather than direct commercial replacements. Future work on formaldehyde emissions, commercial PF benchmarking, durability, and pilot-scale feasibility is also specified.

Location in revised manuscript: Conclusions, pages 38-40, lines 760-786.

Reviewer #2

General reviewer statement: The manuscript addresses the development of lignin–formaldehyde (LF) resins using lignins from different biomass sources and evaluates their performance as wood adhesives. While the topic is relevant in the context of sustainable materials, the manuscript suffers from limited novelty, structural weaknesses, and insufficient critical discussion, which currently reduces its contribution to the field.

No. Comment as provided by reviewer/editor Response

1 Comparing lignins from different biomass sources and their influence on phenol-formaldehyde resin performance is not sufficiently novel. Several prior studies have already demonstrated this topic and yet there was not sufficient review presented in the introduction. Please strengthen the novelty by clearly defining what is genuinely new. The Introduction was revised to distinguish the study from earlier 100% lignin replacement work. The revised novelty is the matched comparison of three unmodified local Pakistani biomass residues across lignin structure, resin processing, curing, and adhesive performance.

Location in revised manuscript: Introduction, pages 4-5, lines 82-90.

2 The manuscript is overly long and repetitive, particularly in:

a. the Abstract that is too dense with numerical results rather than highlighting key findings/trends and overall conclusions

b. Lignin extraction procedures (repeated almost identically for each feedstock)

c. Characterization descriptions (FTIR, TGA, NMR)

d. Result presentation (each lignin and resin discussed separately with similar narrative structure)

I recommend consolidating methods into generic protocols with feedstock-specific deviations summarized in tables, results into comparative sections (CP vs ESD vs SB) instead of sequential descriptions, merge figures where possible rather than separate figures per lignin. The Abstract was revised to emphasize the main trends and interpretation rather than listing excessive numerical details.

The Methods were consolidated into a single generic alkali extraction protocol, with all feedstocks treated under the same conditions.

The FTIR, TGA, NMR, DSC, and lap-shear sections were reorganized into comparative subsections, reducing repeated sample-by-sample narrative.

Figures were reorganized as multi-panel figures. The final figure set includes FTIR, 31P NMR, TGA/DTG, and DSC figures, supplied separately as PLOS-ready TIFF files.

Location in revised manuscript: revised Abstract, pages 2-3, lines 21-52; consolidated extraction method, pages 7, lines 148-153 and 172-176; comparative Results sections, pages 17-19, lines 355-403; merged figure captions, page 21, lines 424-426, page 24, lines 468-472, page 27, lines 548-551, and page 30, lines 595-598.

3 The discussion remains largely descriptive rather than analytical. Key observations are reported but not deeply interpreted. Strengthen discussion by

a. linking lignin structure → reactivity → crosslink density → adhesive performance

b. comparing findings critically with literature (agreement vs deviation)

c. providing mechanistic explanations (e.g., steric hindrance in the different phenolic groups, accessibility of reactive sites, role of molecular weight) The Discussion now explicitly connects lignin source and hydroxyl distribution to methylolation, condensation, crosslink-network development, Tg, gel time, and dry/wet lap-shear strength.

The Discussion was expanded with relevant literature on lignin phenolic adhesives, hydroxyl functionality, molecular weight, and adhesive performance.

Mechanistic interpretation was added, particularly explaining why total hydroxyl abundance alone did not control adhesive performance.

Location in revised manuscript: mechanistic structure-reactivity-performance discussion, pages 24-25, lines 475-491; pages 30-32, lines 601-635; pages 33-34, lines 649-684; and statistical interpretation, pages 36, lines 707-727.

4 The methodology mentions different levels of lignin substitution (0%, 20%, 50%, 80%, 100%), but only one resin per lignin type (apparently at full substitution) is presented in the results, and it´s not clear if this is from a 100% substitution? If it is indeed 100%, the reported viscosity (~0.3 Pa·s at ~50% solids) also seems unusually low, likely due to the very high shear rate (1000 s⁻¹). Although such shear conditions may be relevant for industrial processing, they could mask subtle but potentially important differences arising from formulation changes or lignin type variations especially true for shear thinning phenolic resins. Measurements at lower o

Attachment Submitted filename: Response to Reviewers comments.docx

# Peer review: Decision Letter 1

**DOI:** 10.1371/journal.pone.0352893.r003

**Authors:** Din Bandhu

16 Jun 2026

A lab-scale prototype development of Lignin-Formaldehyde (LF) resin: A bio-renewable adhesive for wood panel industries

PONE-D-26-19567R1

Dear Dr. Hussain,

We’re pleased to inform you that your manuscript has been judged scientifically suitable for publication and will be formally accepted for publication once it meets all outstanding technical requirements.

Within one week, you’ll receive an e-mail detailing the required amendments. When these have been addressed, you’ll receive a formal acceptance letter and your manuscript will be scheduled for publication.

An invoice will be generated when your article is formally accepted. Please note, if your institution has a publishing partnership with PLOS and your article meets the relevant criteria, all or part of your publication costs will be covered. Please make sure your user information is up-to-date by logging into Editorial Manager at Editorial Manager® and clicking the ‘Update My Information' link at the top of the page. For questions related to billing, please contact billing support.

If your institution or institutions have a press office, please notify them about your upcoming paper to help maximize its impact. If they’ll be preparing press materials, please inform our press team as soon as possible -- no later than 48 hours after receiving the formal acceptance. Your manuscript will remain under strict press embargo until 2 pm Eastern Time on the date of publication. For more information, please contact onepress@plos.org.

Kind regards,

Din Bandhu, Ph.D.

Academic Editor

PLOS One

Additional Editor Comments (optional):

Reviewers' comments:

Reviewer's Responses to Questions

**Comments to the Author**

1. If the authors have adequately addressed your comments raised in a previous round of review and you feel that this manuscript is now acceptable for publication, you may indicate that here to bypass the “Comments to the Author” section, enter your conflict of interest statement in the “Confidential to Editor” section, and submit your "Accept" recommendation.

Reviewer #1: All comments have been addressed

Reviewer #3: All comments have been addressed

**********

2. Is the manuscript technically sound, and do the data support the conclusions?

The manuscript must describe a technically sound piece of scientific research with data that supports the conclusions. Experiments must have been conducted rigorously, with appropriate controls, replication, and sample sizes. The conclusions must be drawn appropriately based on the data presented.

Reviewer #1: Yes

Reviewer #3: Yes

**********

3. Has the statistical analysis been performed appropriately and rigorously?

Reviewer #1: (No Response)

Reviewer #3: Yes

**********

4. Have the authors made all data underlying the findings in their manuscript fully available?

The PLOS Data policy requires authors to make all data underlying the findings described in their manuscript fully available without restriction, with rare exception (please refer to the Data Availability Statement in the manuscript PDF file). The data should be provided as part of the manuscript or its supporting information, or deposited to a public repository. For example, in addition to summary statistics, the data points behind means, medians and variance measures should be available. If there are restrictions on publicly sharing data—e.g. participant privacy or use of data from a third party—those must be specified.

Reviewer #1: (No Response)

Reviewer #3: Yes

**********

5. Is the manuscript presented in an intelligible fashion and written in standard English?

PLOS ONE does not copyedit accepted manuscripts, so the language in submitted articles must be clear, correct, and unambiguous. Any typographical or grammatical errors should be corrected at revision, so please note any specific errors here.

Reviewer #1: (No Response)

Reviewer #3: Yes

**********

6. Review Comments to the Author

Please use the space provided to explain your answers to the questions above. You may also include additional comments for the author, including concerns about dual publication, research ethics, or publication ethics. (Please upload your review as an attachment if it exceeds 20,000 characters)

Reviewer #1: (No Response)

Reviewer #3: (No Response)

**********

7. PLOS authors have the option to publish the peer review history of their article (what does this mean?). If published, this will include your full peer review and any attached files.

If you choose “no”, your identity will remain anonymous but your review may still be made public.

**Do you want your identity to be public for this peer review?**  For information about this choice, including consent withdrawal, please see our Privacy Policy.

Reviewer #1: No

Reviewer #3: No

**********

# Editor report: Acceptance letter

**DOI:** 10.1371/journal.pone.0352893.r004

**Authors:** Din Bandhu

PONE-D-26-19567R1

PLOS One

Dear Dr. Hussain,

I'm pleased to inform you that your manuscript has been deemed suitable for publication in PLOS One. Congratulations! Your manuscript is now being handed over to our production team.

At this stage, our production department will prepare your paper for publication. This includes ensuring the following:

* All references, tables, and figures are properly cited

* All relevant supporting information is included in the manuscript submission,

* There are no issues that prevent the paper from being properly typeset

You will receive further instructions from the production team, including instructions on how to review your proof when it is ready. Please keep in mind that we are working through a large volume of accepted articles, so please give us a few days to review your paper and let you know the next and final steps.

Lastly, if your institution or institutions have a press office, please let them know about your upcoming paper now to help maximize its impact. If they'll be preparing press materials, please inform our press team within the next 48 hours. Your manuscript will remain under strict press embargo until 2 pm Eastern Time on the date of publication. For more information, please contact onepress@plos.org.

You will receive an invoice from PLOS for your publication fee after your manuscript has reached the completed accept phase. If you receive an email requesting payment before acceptance or for any other service, this may be a phishing scheme. Learn how to identify phishing emails and protect your accounts at https://explore.plos.org/phishing.

If we can help with anything else, please email us at customercare@plos.org.

Thank you for submitting your work to PLOS ONE and supporting open access.

Kind regards,

PLOS ONE Editorial Office Staff

on behalf of

Dr. Din Bandhu

Academic Editor

PLOS One
